# Monitoring and Risk Assessment of Pesticide Residues in Seafood Using LC-MS/MS

**DOI:** 10.3390/foods14183198

**Published:** 2025-09-13

**Authors:** Dong-ju Kim, Eun-been Oh, Jee-hyo Moon, Jeong-won Choi, Tae-hwa Kim, Seok-hee Lee, Ju-Yeon Park, Chan-Hyeok Kwon, Kee-sung Kyung

**Affiliations:** 1 Department of Environmental and Biological Chemistry, Chungbuk National University, Cheongju 28644, Republic of Korea; kimdj6746@naver.com (D.-j.K.); gsw06059@naver.com (E.-b.O.); myulyng14@gmail.com (J.-h.M.); ww8597@naver.com (J.-w.C.); 2 Analysis Technology and Tomorrow, Daegu 42703, Republic of Korea; thkim@atnt.co.kr; 3 Department of Food Science and Biotechnology, Dongguk University, Goyang 10326, Republic of Korea; seokhee@dongguk.edu; 4 Pesticide and Veterinary Drugs Residues Divisions, National Institute of Food and Drug Safety Evaluation, Ministry of Food and Drug Safety, Osong 28159, Republic of Korea; jy520@korea.kr (J.-Y.P.); chkwon@korea.kr (C.-H.K.)

**Keywords:** monitoring, multi-residue, pesticide, risk assessment, seafood

## Abstract

This study was conducted to monitor 161 pesticides and 37 of their metabolites in cephalopods, crustaceans, seaweeds, and shellfish and to assess their potential risks. A total of 696 types of seafood (227 cephalopods, 56 crustaceans, 189 seaweeds, and 224 shellfish) were collected from local markets across the Republic of Korea and analyzed for pesticide residues using liquid chromatography–tandem mass spectrometry (LC-MS/MS). Pesticide residues were detected only in shrimp among the crustaceans, whereas no residues were found in any species of cephalopods. Pesticide residues in shellfish were detected in abalone, clam, and marsh clam, while in seaweed, they were found in dried laver, dried sea lettuce, and fresh sea mustard. Among seafood products, seaweed had the highest pesticide detection rate of 8.5%, and the residue level of diuron—the most frequently detected insecticide in seaweed—was 0.05 mg/kg. The estimated daily intake (EDI) was calculated using the maximum pesticide concentration in crustaceans, shellfish, cephalopods, and seaweed, along with the seafood consumption by average and the 97.5th-percentile extreme consumers. The percentage of the acceptable daily intake (%ADI), calculated using the EDI and ADI of the pesticide detected, was evaluated to be less than 0.7% for all samples. The results suggest that the consumption of crustaceans, shellfish, cephalopods, and seaweed distributed in the Republic of Korea poses a low risk to human health.

## 1. Introduction

Globally, the production of fisheries and aquaculture products continues to grow, and the fisheries sector is increasingly recognized in the 21st century as essential for global food security and nutrition [1]. Omega-3 polyunsaturated fatty acids (ω-3 PUFA), including eicosapentaenoic acid (EPA) and docosahexaenoic acid (DHA)—which are known to help prevent the onset of depression—are abundant in fish and shellfish [2]. Seaweed has also been reported as a dietary source rich in minerals, proteins, vitamins, and dietary fiber [3]. According to the Ministry of Oceans and Fisheries, the Republic of Korea ranked first among major countries, including Japan, China, the United States, the European Union (EU), etc., in seafood consumption in 2022, with an annual per capita consumption of 68.4 kg [4].

In Korea, dietary patterns have shifted with socioeconomic changes, resulting in greater emphasis on convenience and well-being [5]. Koreans consume a wide variety of seafood, including crustaceans, mollusks, seaweed, and shellfish, prepared in diverse ways such as soups, fermented dishes, stews, braised dishes, and stir-fried dishes [6]. Therefore, the forms and parts of seafood consumed vary depending on the cooking methods and consumer preferences [6]. Pesticides, when bioaccumulated, can be distributed across various parts such as the liver, muscles, fat, and tissues, with the concentration of accumulation varying depending on the properties of the pesticide and the lipid content of the tissues [7,8]. Therefore, it is necessary to conduct a risk assessment based on the analysis of parts of seafood.

The process of bioconcentration in aquatic organisms can largely be divided into the transfer of residual substances to fish that flow into the aquatic environment during rainfall and the consumption of contaminated food [9]. According to data from the Korea Crop Protection Association, a total of 20,746 tons of pesticides were distributed in the Republic of Korea in 2022. Among them, fungicides accounted for the largest proportion at approximately 35%, followed by herbicides at 30%, insecticides at 25%, and other types at 8% [10]. Therefore, even though herbicides are directly applied to the soil, fungicides and insecticides can also leave residue in the soil and have the potential to leach into groundwater [11]. In the case of aquaculture, there is a potential for contamination due to the introduction of unauthorized pesticides sprayed for the purpose of preventing or eliminating pest infections [12]. Additionally, feed is provided for growth and reproduction, and since the raw materials used in the production of feed include agricultural products such as grains, beans, and fishmeal, seafood raised on such feed can accumulate pesticides from the feed [13]. In the case of abalones, juvenile abalones are raised in aquaculture farms where they are fed kelp and sea mustard, and so pesticides with an *n*-octanol/water partition coefficient (log K_ow_) greater than 3 have the potential to bioaccumulate in their body [14,15]. Therefore, it is necessary to monitor and conduct risk assessments of both wild-caught and farmed seafood.

The Codex Alimentarius Commission (CAC) has set the maximum residue limit (MRL) of 0.01 mg/kg for lindane in diadromous fish and marine fish [16]. In the United States, food safety management is divided among several agencies, with the Food and Drug Administration (FDA) publishing the Food Code every four years to maintain consistency in food safety regulations, while the Environmental Protection Agency (EPA) manages pesticide residue limits. The EPA has set residue limits for 26 pesticides, including 2,4-dichlorophenoxyacetic acid, in various aquatic species such as crayfish, crustacean, fish, mollusk, oyster, and shellfish [17]. The Ministry of Health, Labor, and Welfare (MHLW) in Japan manages and sets MRLs for pesticide residues, including both pesticides and veterinary drugs, covering a total of 178 substances. Among foreign countries, Japan has the most comprehensive set of MRL standards for seafood products [18]. The Ministry of Food and Drug Safety (MFDS) in the Republic of Korea has established an MRL only for ethoxyquin in fish and crustaceans [12,19].

This study validated a multi-class-pesticide, multi-residue method for the determination of 161 pesticides and 37 of their metabolites in seaweeds, crustaceans, shellfish, and cephalopods using LC-MS/MS, as suggested by the MFDS [19]. Additionally, for the seaweeds, both fresh and dried samples were analyzed, while for shellfish and cephalopods, muscle, whole, and entire samples were monitored separately. Risk assessment was conducted on the detected samples, with detailed analysis based on various consumption patterns. Finally, monitoring and risk assessment can provide valuable data for evaluating contamination levels of pesticides in seafood, as well as for regulating and managing pesticide residues [20].

## 2. Materials and Methods

### 2.1. Materials

All analytical standards for 161 pesticides and 37 of their metabolites used in the analysis were purchased from AccuStandard (New Haven, CT, USA), Chem Service (West Chester, PA, USA), HPC Standards GmbH (Borsdorf, Germany), Kemidas (Gunpo, Republic of Korea), LGC Standards (Teddington, UK), Sigma-Aldrich (St. Louis, MO, USA), and Wako Pure Chemical (Osaka, Japan). High-performance liquid chromatography (HPLC)-grade acetone, acetonitrile, methanol, and water were sourced from Honeywell (Charlotte, NC, USA). Formic acid (99.0%) was purchased from Samchun (Seoul, Republic of Korea), while ammonium formate (95.0%) and dimethyl sulfoxide (99.0%) were obtained from Kanto Chemical (Tokyo, Japan). The quick, easy, cheap, effective, rugged, and safe (QuEChERS) original kit (4 g of MgSO_4_, 1 g of NaCl) for extraction was purchased from Taesan Science (Gunpo, Republic of Korea), while the dispersive solid-phase extraction (d-SPE) cartridge (150 mg of MgSO_4_, 25 mg of PSA, 25 mg of C18) for purification was obtained from Chiral Technology Korea (Daejeon, Republic of Korea). The 2010 Geno/Grinder from SPEX (Metuchen, NJ, USA) was employed as the extractor, while the centrifuge utilized was the Combi-514R manufactured by Hanil Science Industrial (Gimpo, Republic of Korea). The standards were dissolved in solvents such as acetone, acetonitrile, methanol, or water, depending on their solubility, to prepare stock solutions at concentrations of 100–1000 mg/L. Each stock solution was then used to prepare mixed standard solutions for LC-MS/MS (Shimadzu Corporation, Kyoto, Japan) analysis, which were stored frozen at –20 °C until analysis.

### 2.2. Sample Selection and Collection

For the analysis of pesticide residues in seafood, a total of 13 seafood species, including seaweed and invertebrates such as crustaceans and mollusks, were selected based on the high consumption rates reported in the Korea Health Industry Development Institute (KHIDI) [21], the domestic pesticide-detection history [22,23], and species with high seasonal consumption. In 2024, among the collected species, mollusks were further classified into shellfish and cephalopods, and three additional cephalopod species with high consumption rates were selected that year. The selected types of seafood by species are presented in Table 1, and their names were referenced from the Food Code [24]. For nationwide sample collection, the Republic of Korea was divided into nine regions proportional to the population size and population density of administrative districts based on Statistics Korea data. Sampling areas were selected in order of regions with high population density: Seoul, Gyeonggi/Incheon, Busan, Daegu/Ulsan, Gangwon, Chungcheong/Daejeon/Sejong, Gyeongsang, Jeju, and Jeolla/Gwangju. Additionally, while the Republic of Korea’s seafood market operates through diverse distribution channels, samples were collected to ensure representativeness by reflecting the main channels through which consumers primarily purchase seafood, such as wholesale markets, large-scale supermarkets, and traditional markets.

A total of 504 seafood samples distributed in the Republic of Korea were purchased over a two-year period from May 2023 to November 2024. The types of seafood collected included 56 crustaceans, 189 seaweeds, 160 shellfish, and 99 cephalopods. Of the total 504 samples, 175 samples were wild-caught, and 329 samples were farmed, with a ratio of 35:65. Domestic products accounted for 434 samples, while 70 were imported, with a ratio of 86:14. Among the imported samples, China accounted for the largest number with 25 cases; followed by Ecuador (15 cases); Russia (11 cases); distant-water sources (9 cases); Thailand (4 cases); Vietnam (3 cases); and Malaysia, the Atlantic, and Chile (1 case each). The collected samples were individually packaged by species to prevent contamination, damage, thawing, or deformation and were transported to the laboratory on the same day. The raw materials of the seafood for analysis were prepared according to the guidelines of the MFDS [25], with only the edible parts being collected as a principle to ensure a minimum quantity of at least 1 kg. The collected samples were classified as cephalopods (muscle parts with internal organs and eyes removed), crustaceans (muscle parts with shells, heads, tails, and legs removed), seaweeds (whole, either dried or fresh), and shellfish (muscle parts with shells and internal organs removed). Additionally, in 2024, based on consumption trends, the analysis for cephalopods, such as squid and octopus, and shellfish, such as abalone, included not only the existing muscle parts but also the internal organs and the entire body.

**Figure 1:**
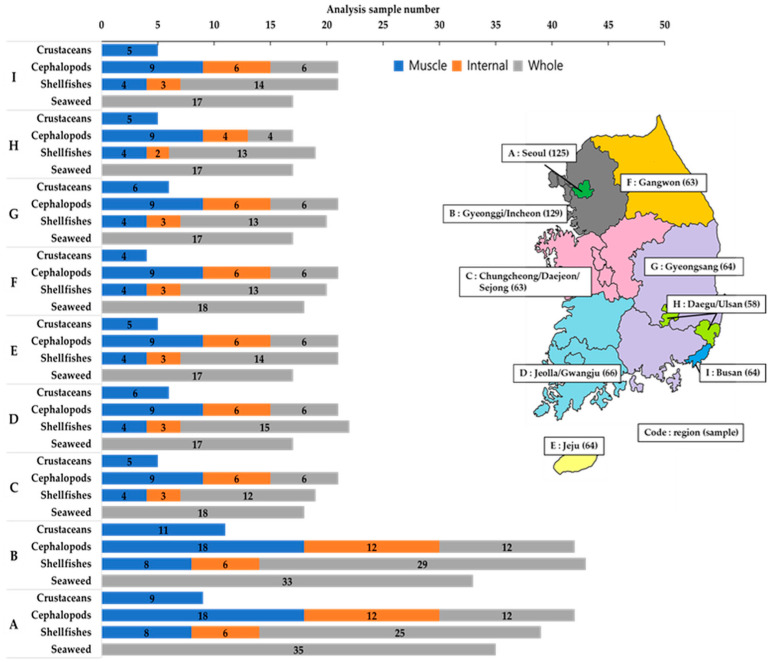
Regional distribution by body part (muscle, internal, and whole). Values in parentheses indicate the number of samples collected.

As a result, the analysis samples were allocated as follows: 56 samples for crustaceans, 189 samples for seaweeds, 224 samples for shellfish (149 for whole-body, 43 for muscle, and 32 for internal organs), and 227 samples for cephalopods (99 for whole-body, 64 for muscle, and 64 for internal organs). In total, 696 samples were used for the analysis. The regional distribution of analysis points is presented in Figure 1. The samples were transported to the laboratory on the same day in insulated containers with ice packs to maintain a low temperature, and then processed by separating the parts for analysis, freezing them, homogenizing them with dry ice, and storing them in a freezer at −20 °C until analysis.

### 2.3. Analysis

In this study, pesticide residues in the samples were analyzed using a multi-class-pesticide, multi-residue method based on the QuEChERS pretreatment method proposed by MFDS [19]. For crustaceans and mollusks (shellfish and cephalopods), 5 g of homogenized sample was accurately weighed into a 50 mL centrifuge tube, followed by the addition of 10 mL of acetonitrile. The mixture was shaken at 1300 rpm for 1 min. For seaweeds, 15 g of fresh sample or 2 g of dried sample (with 10 mL of water added to rehydrate the dried sample) was accurately weighed into a 50 mL centrifuge tube. Fresh samples were mixed with 20 mL of acetonitrile, and dried samples were mixed with 10 mL of acetonitrile, followed by shaking at 1300 rpm for 1 min. Subsequently, 4 g of MgSO_4_ and 1 g of NaCl were added, and the mixture was shaken at 1300 rpm for 10 min before being centrifuged at 3000 rpm and 4 °C for 10 min. After centrifugation, 1 mL of the supernatant was transferred to a 2 mL centrifuge tube containing 150 mg of MgSO_4_, 25 mg of C18, and 25 mg of primary secondary amine (PSA). The mixture was shaken for 1 min and then centrifuged at 10,000 rpm and 4 °C for 2 min. The supernatant was then filtered through a 0.2 μm PTFE membrane filter and used as the test solution. The analysis was performed using a Shimadzu LC-MS/MS-8050 triple quadrupole mass spectrometer (Shimadzu Corporation, Kyoto, Japan) in conjunction with a Nexera liquid chromatograph (Shimadzu Corporation, Kyoto, Japan). The liquid chromatograph system comprised a column oven (CTO-40C), an autosampler (SIL-40CX3), and a pump (LC-40BX3). Pesticide separation was achieved using a Phenomenex Kinetex C18 column (150 mm L × 2.1 mm I.D., 2.6 μm particle size; California, USA). The column oven temperature was maintained at 40 °C, with a flow rate of 0.4 mL/min and an injection volume of 5 μL. The mobile phase consisted of 1 mM ammonium formate with 0.1% formic acid in water (A) and 1 mM ammonium formate with 0.1% formic acid in methanol (B). The gradient elution program for mobile phase B was as follows: 10% for 1.0 min, ramped to 55% over the next 3.0 min, ramped to 100% over the next 7.5 min, held at 100% for 1.5 min, then decreased back to 10% over 0.01 min and equilibrated for an additional 2.99 min. Positive and negative electrospray ionization (ESI^+^/ESI^−^) and multiple reaction monitoring (MRM) were employed to analyze the target pesticides. Argon (purity 99.999%) was used as the collision-induced dissociation (CID) gas. The desolvation line (DL) and heat block temperatures were maintained at 250 °C and 400 °C, respectively. The drying and nebulizing gas flow rates were both set at 10.0 L/min. Data processing was performed using Shimadzu LabSolutions software version 5.98. To obtain MRM transition profiles, a full scan mode was employed to scan the mass-to-charge ratio (*m*/*z*) from 150 to 800 at a rate of 410 u/s. For each pesticide, the precursor ion was selected from the scan spectrum and subjected to a product ion scan at varying collision energies (CEs) to confirm its fragmentation pattern. The selection of the quantifier and qualifier ions under the optimal CE was based on their sensitivity and selectivity. The MRM conditions for the 198 compounds, including 161 pesticides, are provided in Table S1.

### 2.4. Method Validation

The validity of the multi-class-pesticide, multi-residue method for pesticide residues in seafood was verified based on the Codex Guidelines (CAC/GL 71) [26]. Clam, shrimp, squid, dried laver (dried gim), and fresh laver (fresh gim) were selected as representative seafood samples for shellfish, crustaceans, cephalopods, and seaweeds, respectively, based on their high intake rates. Representative samples without detectable pesticide residues were used to evaluate the linearity, mean recovery, and coefficient of variation (CV) of the analytical method, assessing its linearity, accuracy, and precision.

To minimize the impact of the sample matrix, matrix-matched calibration was used, achieving a matrix adjustment ratio of 90%. To confirm the linearity of matrix-matched standard solutions, mixed standard solutions were diluted with untreated sample extracts to create calibration curves within a concentration range of 0.001–0.1 mg/L using peak areas. The coefficient of determination (R^2^) was verified to be at least 0.98. The limits of detection (LODs) and limits of quantification (LOQs) for all compounds were established based on signal-to-noise (S/N) ratios of 3 and 10, respectively. The LOQs for all compounds were 0.01 mg/kg. For the evaluation of accuracy and precision, untreated samples were spiked with mixed standard solutions at LOQ, 2× LOQ, and 10× LOQ levels, with five replicates for each concentration. The mean recovery and CVs of the target compounds were verified to meet the criteria of 60–120% and ≤30% at the LOQ, and 70–120% and ≤20% at 2× LOQ and 10× LOQ.

To assess the matrix effect (ME) for each seafood, the slopes of the standard solution calibration curves prepared in pure solvent were compared to the slopes of the matrix-matched calibration curves for each representative sample (clam, dried seaweed, fresh seaweed, shrimp, and squid). The matrix effect for each compound was calculated using Equation (1). The calculated matrix effect values were categorized as soft (ranging from −20% to 20%), medium (ranging from −50% to −20% or from 20% to 50%), and strong (less than −50% or greater than 50%) [27,28].

(1) Matrix effect (%) = (Slope of calibration of matrix/slope of calibration in solvent − 1) × 100

### 2.5. Risk Assessment

Based on the results of the residue monitoring, a risk assessment was conducted using food consumption data and the detected concentrations, following the guidelines for preparing risk assessment reports by the MFDS [29]. Using data from the 8th Korea National Health and Nutrition Examination Survey (2019–2021) [21], the daily food intake of the population was analyzed, and the estimated daily intake (EDI) for each pesticide was calculated based on the maximum residue of detected pesticides and the average body weight of the population. At this time, daily food intake was divided into total consumers and target consumers based on seafood consumption and calculated as the average intake and extreme intake (97.5th percentile). For seafood with no consumption data, the intake was estimated based on samples in a similar category. The average body weight of total consumer was 60 kg, while the average body weight of target consumer was determined based on the average body weight of target consumers from the 8th Korea National Health and Nutrition Examination Survey (2019–2021). The acceptable daily intake (ADI) values for the detected substances were obtained from the MFDS [30], Rural Development Administration (RDA) [31], European Food Safety Authority (EFSA) [32], EPA [33], World Health Organization (WHO) [34], and the Food Safety Commission of Japan (FSCJ) [35] and these values are presented in Supplementary Table S5. The risk of pesticide residues in different types of seafood at the distribution stage was assessed as a risk percentage (%) based on the reported ADI values (Equations (2)–(4)). According to the Common Guidelines for the Risk Assessment of Consumer Products, a %ADI value of 100 or more is considered hazardous, while a value of 100 or less is deemed safe [36].

(2) ADI (mg/kg/day) = ADI (mg/kg·BW/day) of test pesticide × average body weight

(3) EDI (mg/kg/day) = Detected pesticide concentration (mg/kg) × food intake (kg/day)/BW (kg)

(4) %ADI = EDI (mg/kg/day)/ADI (mg/kg/day) × 100

## 3. Results and Discussion

### 3.1. Method Validation

**Figure 2:**
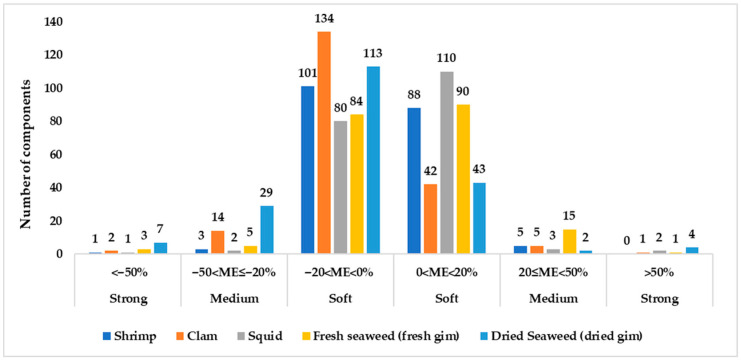
Matrix effect of clam, dried laver, fresh laver, shrimp, and squid. The calculated ME values were categorized as soft (−20% to 20%), medium (−50% to −20% or 20% to 50%), and strong (<−50% or >50%) according to Equation (1).

The linearity of matrix-matched standard solutions was evaluated using the coefficient of determination (R^2^), which was found to be above 0.98 for clam, dried laver, fresh laver, shrimp, and squid. The number of compounds that met the recovery range and precision requirements specified by the Codex Guidelines (CAC/GL 40) was 190 for squid, 194 for shrimp, 195 for clam, 193 for fresh laver, and 180 for dried laver, as summarized in Table S2. The Association of Official Analytical Chemists (AOAC) Food Matrix Triangle classifies food matrices based on their fat, protein, and carbohydrate content [37], and the matrix compounds of representative seafood samples are summarized in Table 2 [38]. In particular, dried seaweed, which contains abundant dietary fiber and pigments, poses limitations in the analysis of certain compounds due to interference from the matrix. Additionally, dried samples tend to exhibit relatively strong adsorption of nonpolar pesticides, which may reduce the extraction efficiency [39]. Such matrices contain thousands of compound substances that can interfere with the ionization efficiency of analytes through various mechanisms, including ion charge competition and changes in electrospray droplet formation [40]. As a result of the matrix effect evaluation, the number of compounds falling within the soft range was 176 (88.9%) for clam, 189 (95.5%) for shrimp, 190 (96.0%) for squid, 156 (78.8%) for dried laver, and 174 (87.9%) for fresh laver, as summarized in Figure 2 and Table S3. While the soft range indicates negligible matrix effects that can be ignored, quantitative analysis must account for matrix effects by mitigating them for compounds classified in the median and strong ranges for each representative sample [28]. In this study, a matrix-matched method was chosen to minimize the impact of matrix effects for seafood and target pesticides and to reduce errors caused by the matrix. The matrix-matched method is suitable for overcoming matrix effects in LC-MS/MS and generating accurate results [41].

As a result of validating the multi-class-pesticide, multi-residue method for pesticides in seafood, it was confirmed that the method could be applied to 190 compounds in squid, 194 compounds in shrimp, 195 compounds in clam, 193 compounds in fresh laver, and 180 compounds in dried laver. Subsequently, the residue levels in seafood samples distributed in the Republic of Korea were investigated.

### 3.2. Monitoring Results of Fishery Products

**Figure 3:**
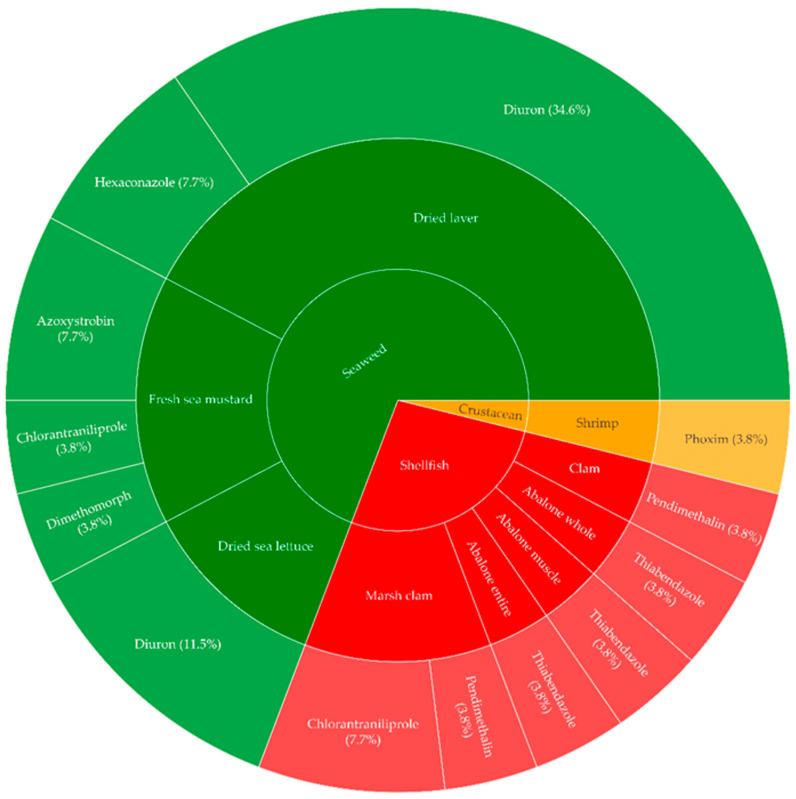
Detection rates of pesticide residues in crustacean, seaweed, and shellfish.

The monitoring results of 56 crustaceans, 224 shellfish, 189 seaweeds, and 227 cephalopods purchased in 2023 and 2024 are presented in Table 3 and Figure 3. All cephalopods showed no detections. In the Republic of Korea, maximum residue limits (MRLs) are currently established only for ethoxyquin in fishery products. None of the 696 samples analyzed in this study exceeded this MRL. For other detected pesticides, no Korean MRLs have been established for fishery products, therefore, exceedance could not be assessed.

The pesticide detected in shrimp among the crustaceans was phoxim, with a concentration of 0.02 mg/kg. Among the shellfish, chlorantraniliprole and pendimethalin were detected in marsh clams, while only pendimethalin was detected in clams. In abalone, thiabendazole was detected at 0.01 mg/kg in the muscle and whole samples, while thiabendazole (0.02 mg/kg) and its metabolite, 5-hydroxy thiabendazole (0.01 mg/kg), were detected in the internal samples. An analysis of seawater and sediment from the southern coast of the Republic of Korea detected maximum residues of thiabendazole at 2.84 ng/L and 0.24 mg/kg, respectively [42]. As 99% of abalone farming in the Republic of Korea occurs along the southern coast [43], it is considered that thiabendazole was detected in abalone due to this region. The CYP450 enzyme present in abalone metabolizes thiabendazole into its major metabolite, 5-hydroxy thiabendazole, as 5-hydroxy thiabendazole was detected in the internal organs of the abalone [44,45].

Pesticides were detected in the seaweeds, including dried laver, dried sea lettuce, dried sea mustard, and fresh sea mustard. Diuron is an antifouling biocide used on marine vessels to remove organisms such as bacteria, diatoms, macroalgae, tunicates, barnacles, mussels, or tubeworms from submerged surfaces in seawater [46]. Diuron has a long aquatic half-life of 43 days and has been detected in the Republic of Korea, Japan, and southern Florida, USA [47]. Diuron was detected at 0.01 mg/kg in shrimp samples collected from the eastern coast of China, and 0.49 µg/kg was detected in oysters from Australia [48,49]. Therefore, since diuron has been detected in seawater, it can be concluded that the concentrations of diuron in dried laver and dried sea lettuce are 0.01–0.05 mg/kg and 0.01–0.02 mg/kg, respectively. Azoxystrobin was detected in fresh sea mustard at concentrations of 0.02–0.03 mg/kg, which is lower than the MRL of 0.08 mg/kg for seafood in Japan. Additionally, chlorantraniliprole and dimethomorph were detected in fresh sea mustard at 0.01 mg/kg and 0.03 mg/kg, respectively, while hexaconazole was detected in dried laver at concentrations of 0.02–0.03 mg/kg. Azoxystrobin, chlorantraniliprole, dimethomorph, and hexaconazole were detected in rivers in the Republic of Korea during August and September. The detection of these pesticides is likely due to residual pesticides from rice paddies, which may have either drifted directly into the river water or been transferred to the river through the discharge of water remaining in the soil. The river water then flows into the sea, resulting in the detection of pesticides in seaweeds [22].

### 3.3. Risk Assessment for Average Consumers and Extreme Consumers

This study investigated 696 seafood samples distributed in the Republic of Korea, analyzing validated compounds in each representative sample. The risk assessment was conducted by dividing consumers into two groups—the average consumers and extreme consumers—and evaluating the risk. The results are summarized in Table 4.

The monitoring results showed that a total of eight pesticides were detected in 696 types of seafood. Five of these pesticides, excluding phoxim, thiabendazole, and pendimethalin, were detected in seaweed (dried laver, dried sea lettuce, and fresh sea mustard). Risk assessment results for the average consumer showed that diuron was detected at 0.04% in dried laver and 0.01% in dried sea lettuce, while azoxystrobin, chlorantraniliprole, dimethomorph, and hexaconazole were all below 0.01% in fresh sea mustard and dried laver. Chlorantraniliprole, thiabendazole, and pendimethalin were detected in shellfish, including clam, abalone (whole, internal, and muscle), while phoxim was detected in crustaceans, specifically shrimp. Since there is no consumption data available for specific abalone parts, the risk assessment was conducted based on the total consumption of whole abalone. The results show a maximum of 0.02%. This assumption may lead to over- or under-estimation of dietary exposure.

On the other hand, seafood consumption is significantly influenced by factors such as age, gender, region, presence of children, and the age of children, resulting in markedly different consumer preferences compared to agricultural and livestock products. Therefore, risk assessments based on the average consumption data for the general population may underestimate the risks for specific groups of high-seafood consumers. To address this, an additional risk assessment was conducted using the extreme consumption (97.5th percentile) of seafood consumers. As a result, diuron was detected at levels of 0.13% in dried laver and 0.66% in dried sea lettuce, while azoxystrobin, chlorantraniliprole, dimethomorph, and hexaconazole were all found at levels below 0.03% in fresh sea mustard and dried laver. In shellfish and crustaceans, chlorantraniliprole, thiabendazole, pendimethalin, and phoxim were detected at levels ranging from 0.00% to 0.19%. Previous studies, which considered worst-case scenarios based on the 97.5th-percentile consumption and the maximum detected pesticide levels, also reported that all pesticide residues were below 2% and thus safe. Similarly, the risk assessment conducted in this study for extreme consumers confirmed that seafood consumption in the Republic of Korea is very safe. Although cumulative risk (HI/MOET) was not quantitatively assessed in this study, the %ADI values for individual pesticides were all below 1%, indicating that the cumulative dietary risk is also likely to be negligible. These findings indicate that pesticide residue levels in seafood distributed in the Republic of Korea are well-managed and at a safe level for the Korean population.

## 4. Conclusions

This study was conducted to validate the multi-class-pesticide, multi-residue method for the analysis of 161 pesticides and 37 of their metabolites in different types of seafood, as suggested by the MFDS, to monitor pesticide residues and to conduct a risk assessment. Seafood sampling in 2023 focused on crustaceans, shellfish, and seaweeds, which have higher consumption rates. In 2024, cephalopods were added to the sampling. A total of 696 samples were collected from nine regions across the country, including 56 samples from two crustacean species, 224 samples from six shellfish species, 227 samples from three cephalopod species, and 189 samples from five seaweed species. As a result of monitoring, phoxim was detected in one shrimp sample among the crustaceans, while no detections were observed in any of the cephalopod samples. Pesticide residues of chlorantraniliprole, pendimethalin, and thiabendazole were detected in shellfish samples, including abalone (whole, muscle, and internal), clam, and marsh clam. Azoxystrobin, chlorantraniliprole, dimethomorph, hexaconazole, and diuron were detected in seaweed samples such as fresh sea mustard, dried sea lettuce, and dried laver. Among the seaweeds, diuron was most frequently detected in dried laver. Seaweeds also showed the highest detection rate (8.5%), with diuron being the most frequently detected pesticide. All pesticides detected in the different types of seafood did not exceed 0.05 mg/kg; however, pesticide residues are consistently detected in seafood, even at low levels, suggesting the need for continued surveillance, particularly in seaweeds. The detection of pesticides in seafood is considered to be due to substances used as antifouling biocides on ships and unintentional contamination from pesticides used in agricultural environments. The risk assessment of different types of seafood for average and 97.5th-percentile extreme consumers showed that the %ADI was below 0.7% for all, indicating that consuming distributed crustaceans, shellfish, seaweeds, and cephalopods is safe for consumers. Additionally, since pesticide levels tend to decrease during washing and cooking, the actual pesticide risk to consumers is likely to be lower. These findings highlight the importance of the continuous monitoring of pesticide residues in fishery products and can serve as a scientific basis for future risk management strategies and regulatory policies to ensure food safety. Similarly, a recent regional seafood monitoring study [20] reported low-level detections of pesticides, such as oxadiazon in littleneck clams, with %ADI values well below health concern thresholds. Both studies consistently show that pesticide residues in seafood are present at low concentrations and pose negligible dietary risks, underscoring the importance of continued monitoring, particularly for high-consumption commodities such as seaweed.

## Figures and Tables

**Table 1:** Fishery products collected to determine the characteristics of pesticide residues in aquatic products and to assess their potential risks in this study.

Category	Sub Category	Fishery Product (Sampling Number)
Invertebrate	Crustacean	Crab (12), shrimp (44)
	Mollusk	(1) Shellfish: abalone whole (32), abalone muscle (44), abalone internal (32), blood cockle (8), clam (44), marsh clam (12), mussel (44), oyster (8)(2) Cephalopod: long-arm octopus whole ^(a)^ (32), long-arm octopus muscle ^(a)^ (33), long-arm octopus internal ^(a)^ (32), octopus^(a)^ (33), squid whole ^(a)^ (32), squid muscle ^(a)^ (33), squid internal ^(a)^ (32)
Algae	Seaweed	Kelp (10), laver (8), sea lettuce (13), sea mustard (13), seaweed furcate (4), dried kelp (34), dried laver (36), dried sea lettuce (31), dried sea mustard (31), dried seaweed furcate (9)

^(a)^ Additional species for analysis in 2024.

**Table 2:** Matrix composition of fishery products.

Fishery Product	Matrix Composition			
	Carbohydrates (%)	Fat (%)	Protein (%)	Moisture (%)
Clam	2.3	1.2	12.5	81.5
Dried laver	42.9	2.3	37.8	5.5
Fresh laver	2.0	0.4	3.3	90.5
Shrimp	1.7	0.9	21.2	75.9
Squid	1.0	0.6	15.0	81.6

**Table 3:** Concentrations of eight detected compounds in fishery products.

Pesticide	Fishery Product		Sample Size	Detection Number			Detection Rate (%)	Concentration (mg/kg)		MRL ^(a)^ (mg/kg)
				Total	Domestic	Imported		Range	Mean	
Azoxystrobin	Seaweed	Fresh sea mustard	13	2	2	-	15.4	0.02–0.03	0.03	Japan: 0.08 (seafood)
Chlorantraniliprole	Seaweed	Fresh sea mustard	13	1	1	-	7.7	0.01	0.01	-
	Shellfish	Marsh clam	12	2	1	1	16.7	0.01–0.03	0.02	-
Dimethomorph	Seaweed	Fresh sea mustard	13	1	1	-	7.7	0.03	0.03	-
Diuron	Seaweed	Dried laver Dried sea lettuce	36 31	9 3	9 3	- -	25.0 9.7	0.01–0.05 0.01–0.02	0.02 0.01	-
Hexaconazole	Seaweed	Dried laver	36	2	2	-	5.6	0.02–0.03	0.03	-
Pendimethalin	Shellfish	Clam Marsh clam	44 12	1 1	1 1	- -	2.3 8.3	0.02 0.02	0.02 0.02	Japan: 0.3 (seafood)
Phoxim	Crustacean	Shrimp	44	1	-	1	2.3	0.02	0.02	-
Thiabendazole	Shellfish	Abalone internal Abalone muscle Abalone whole	32 44 32	1 1 1	1 1 1	- - -	3.1 2.3 +3.1	0.03 0.01 0.01	0.03 0.01 0.01	Japan: 0.02 (shellfish)

^(a)^ MRL: maximum residue limit.

**Table 4:** Assessment of dietary exposure to the detected pesticides for average and extreme consumption.

Pesticide	ADI ^(a)^ (mg/kg·BW/day)	Species of Seafood	Average Consumption					Extreme Consumption				
			IR ^(b)^ (kg/day)	MC ^(c)^ (mg/kg)	BW ^(d)^ (kg)	EDI ^(e)^ (mg/kg· BW/day)	%ADI	IR (kg/day)	MC (mg/kg)	BW (kg)	EDI (mg/kg· BW/day)	%ADI
Azoxystrobin	0.2	Fresh sea mustard	0.0008	0.03	60.0	3.8 × 10^−7^	0.00	0.0053	0.03	58.2	2.6 × 10^−6^	0.00
Chlorantraniliprole	2	Fresh sea mustard	0.0008	0.01		1.3 × 10^−7^	0.00	0.0053	0.01	58.2	8.8 × 10^−7^	0.00
		Marsh clam	0.0008	0.03		3.8 × 10^−7^	0.00	0.0653	0.03	59.7	3.3 × 10^−5^	0.00
Dimethomorph	0.2	Fresh sea mustard	0.0008	0.03		3.8 × 10^−7^	0.00	0.0053	0.03	58.2	2.6 × 10^−6^	0.00
Diuron	0.002	Dried laver Dried sea lettuce	0.0010 0.0004	0.05 0.02		8.5 × 10^−7^ 1.3 × 10^−7^	0.04 0.01	0.0032 0.0396	0.05 0.02	58.6 62.6	2.6 × 10^−6^ 1.3 × 10^−5^	0.13 0.66
Hexaconazole	0.005	Dried laver	0.0010	0.03		5.1 × 10^−7^	0.01	0.0032	0.03	58.6	1.6 × 10^−6^	0.03
Pendimethalin	0.13	Clam Marsh clam	0.0010 0.0008	0.02 0.02		3.4 × 10^−7^ 2.5 × 10^−7^	0.00 0.00	0.0117 0.0653	0.02 0.02	60.2 59.7	3.9 × 10^−6^ 2.2 × 10^−5^	0.00 0.02
Phoxim	0.004	Shrimp	0.0028	0.02		9.5 × 10^−7^	0.02	0.0233	0.02	59.8	7.8 × 10^−6^	0.19
Thiabendazole	0.1	Abalone internal Abalone muscle Abalone whole	0.0005	0.03 0.01 0.01		2.4 × 10^−7^ 8.1 × 10^−8^ 8.1 × 10^−8^	0.00 0.00 0.00	0.0224	0.03 0.01 0.01	62.1	1.1 × 10^−5^ 3.7 × 10^−6^ 3.7 × 10^−6^	0.01 0.00 0.00

^(a)^ Acceptable daily intake; ^(b)^ IR: intake rate; ^(c)^ MC: maximum concentration; ^(d)^ BW: body weight; ^(e)^ estimated daily intake.

## Data Availability

The original contributions presented in this study are included in the article/Supplementary Materials. Further inquiries can be directed to the corresponding author.

## Supplementary Materials

The following supporting information can be downloaded at: https://www.mdpi.com/article/10.3390/foods14183198/s1, **Table S1.** Multiple reaction monitoring (MRM) transitions used for method validation of pesticide residues by LC–MS/MS; **Table S2.** Coefficients of determination (R^2^) of calibration curves for all target compounds; **Table S3.** Recoveries and coefficients of variation (CVs) of 161 pesticides and 37 of their metabolites at three concentrations using the method suggested by MFDS; **Table S4.** Matrix effects on the 198 compounds in shrimp, manila clam, laver, dried laver, and squid; **Table S5.** Acceptable daily intake (ADI) values for all analyzed compounds.
