# Supplementary material for: Monitoring and Risk Assessment of Pesticide Residues in Seafood Using LC-MS/MS
Source: Foods. 2025 Sep 13;14(18):3198. doi: 10.3390/foods14183198 (PMC12469867; doi:10.3390/foods14183198)
Supplement: Supplementary file 1 [file foods-14-03198-s001.zip › foods-3853013-supplementary.pdf]

Supplementary material

Table S1. Multiple reaction monitoring (MRM) transitions used for method validation of pesticide residues by LC–MS/MS

No.	Pesticide and metabolite	Ionization	Retention time (min)	Precursor ion (m/z)	Quantitative ion (m/z)	Collision Energy (eV)	Qualitative ion (m/z)	Collision Energy (eV)
1	Acephate	+	1.93	184.0	143.0	-10.0	95.2	-24.0
2	Acetamiprid	+	3.96	223.1	126.2	-21.0	99.1	-38.0
	N-Desmethyl-acetamiprid (IM-2-1)	+	3.97	209.0	126.0	-16.0	99.0	-37.0
3	Acynonapyr	+	10.35	505.1	342.0	-16.0	122.1	-30.0
4	Alachlor	+	6.81	270.2	162.2	-20.0	132.1	-39.0
5	Aldicarb	+	4.36	208.1	89.0	-16.0	116.1	-8.0
6	Amitraz	+	9.46	294.1	163.1	-20.0	122.1	-30.0
	2,4-Dimethylaniline	+	3.36	122.2	107.1	-20.0	77.0	-29.0
7	Atrazine	+	5.40	216.2	174.1	-17.0	96.1	-25.0
	Atrazine-desethyl (DEA)	+	4.18	188.2	146.1	-17.0	79.0	-26.0
	Atrazine-desisopropyl (DIA)	+	3.68	174.0	68.1	-28.0	79.1	-18.0
8	Azinphos-methyl	+	5.72	317.9	132.0	-13.0	159.9	-7.0
9	Azoxystrobin	+	5.90	404.1	372.2	-25.0	344.2	-35.0
10	Bendiocarb	+	4.72	224.1	109.2	-16.0	167.1	-9.0
11	Bensulfuron methyl	+	5.71	411.1	149.1	-21.0	182.0	-21.0
12	Benzovindiflupyr	+	7.37	398.0	342.1	-19.0	378.1	-15.0
13	Benzpyrimoxan	+	7.05	341.0	109.0	-50.0	87.1	-26.0
14	Bifenazate	+	6.56	301.2	170.2	-21.0	152.1	-42.0
	Bifenazate-diazene	+	8.08	299.1	197.1	-20.0	196.1	-15.0
15	Bioresmethrin	+	9.98	339.1	171.1	-16.0	128.1	-39.0
16	Boscalid	+	7.47	344.1	161.0	-25.0	69.0	-22.0
17	Brodifacoum	+	9.98	523.0	335.0	-22.0	178.1	-33.0
18	Buprofezine	+	8.60	306.1	57.1	-15.0	116.1	-20.0
19	Butamifos	+	7.73	333.2	152.1	-15.0	180.1	-15.0
20	Cafenstrole	+	6.49	351.0	100.2	-12.0	72.1	-27.0
21	Carbaryl	+	4.92	202.1	127.2	-25.0	145.2	-13.0
22	Carbendazim	+	3.36	192.0	132.1	-30.0	105.1	-34.0
23	Carbofuran	+	4.75	222.0	123.1	-21.0	165.2	-12.0
	3-Hydroxycarbofuran	+	3.96	238.0	181.1	-12.0	163.2	-20.0
24	Carbosulfan	+	10.18	381.3	76.1	-35.0	160.1	-16.0
25	Carfentrazone-ethyl	+	7.21	412.1	346.0	-24.0	383.9	-15.0
26	Carpropamide	+	7.45	334.0	139.0	-21.0	103.1	-42.0
27	Chlorantraniliprole	+	5.68	482.0	450.9	-17.0	112.0	-55.0
28	Chlorfenvinphos *E*	+	7.48	358.9	99.1	-33.0	155.2	-14.0

	Chlorfenvinphos *Z*	+	7.48	358.9	99.1	-33.0	155.2	-14.0
29	Chromafenozide	+	6.70	395.2	175.2	-18.0	147.1	-44.0
30	Clofentezine	+	7.81	303.0	138.0	-16.0	102.2	-36.0
31	Clomeprop	+	8.54	324.0	120.1	-21.0	203.0	-15.0
32	Clothianidin	+	3.80	250.0	169.1	-13.0	132.1	-16.0
33	Cumyluron	+	6.52	303.1	119.3	-21.0	73.1	-19.0
34	Cyantraniliprole	+	4.99	474.8	285.9	-16.0	443.9	-21.0
35	Cyclopyrimorate	+	6.39	390.1	70.0	-26.0	114.1	-15.0
36	Cyproconazole	+	6.49	292.1	70.2	-18.0	125.2	-30.0
37	Daimuron	+	6.40	269.1	151.2	-12.0	119.1	-21.0
38	Diazinon	+	7.44	305.2	100.0	-40.0	84.1	-34.0
39	Dichlorvos (DDVP)	+	4.66	239.0	182.2	-19.0	85.1	-16.0
40	Diclocymet *E*	+	7.03	313.1	137.0	-30.0	173.0	-17.0
	Diclocymet *Z*	+	7.03	313.1	137.0	-30.0	173.0	-17.0
41	Diflubenzuron	+	6.88	311.1	158.0	-16.0	141.0	-25.0
42	Dimethomorph *E*	+	6.06	388.1	301.1	-21.0	273.2	-15.0
	Dimethomorph *Z*	+	6.06	388.1	301.1	-21.0	273.2	-15.0
43	Dinotefuran	+	2.83	203.1	129.2	-13.0	113.2	-12.0
44	Disulfoton	+	4.07	275.0	61.1	-48.0	29.1	-52.0
	Disulfoton sulfone	+	5.21	307.0	125.0	-17.0	153.0	-12.0
	Disulfoton sulfoxide	+	5.15	291.0	185.1	-14.0	213.1	-10.0
	Demeton-S	+	5.82	259.0	89.1	-15.0	61.0	-32.0
	Demeton-S-sulfone	+	4.11	291.0	235.1	-15.0	263.2	-12.0
	Demeton-S-sulfoxide	+	4.06	275.1	81.1	-39.0	169.0	-17.0
45	Diuron	+	5.48	233.0	72.1	-22.0	46.1	-17.0
46	Edifenphos	+	7.37	311.0	109.0	-26.0	283.0	-19.0
47	Emamectin benzoate	+	8.65	886.3	158.2	-34.0	126.2	-40.0
48	Epoxyconazole	+	6.80	330.0	121.1	-21.0	123.0	-17.0
49	Esprocarb	+	8.55	266.2	71.1	-15.0	65.0	-54.0
50	Ethiofencarb	+	5.05	226.2	107.1	-15.0	164.1	-8.0
51	Ethiprole	+	6.01	397.1	350.9	-21.0	227.9	-52.0
	Ethiprole-sulfone	-	6.02	411.1	282.0	28.0	244.0	45.0
52	Ethoxyquin	+	5.73	218.1	174.1	-19.0	190.1	-18.0
	Ethoxyquin dimer	+	10.90	433.3	188.1	-42.0	375.1	-33.0
53	Etobenzanid	+	7.44	340.1	121.1	-34.0	149.1	-22.0
54	Etoxazole	+	9.26	360.2	304.2	-15.0	113.0	-35.0
55	Etrimfos	+	7.41	293.0	265.1	-17.0	125.1	-20.0
56	Famoxadone	+	7.51	392.1	331.2	-11.0	238.1	-17.0
57	Fenamidone	+	6.09	312.1	65.1	-49.0	92.1	-26.0
58	Fenarimol	+	6.74	331.1	268.0	-22.0	258.9	-25.0
59	Fenbuconazole	+	6.91	337.1	125.1	-29.0	194.1	-17.0
60	Fenhexamid	+	6.66	301.9	97.1	-24.0	55.1	-41.0

61	Fenobucarb	+	5.89	208.1	77.1	-38.0	95.1	-14.0
62	Fenoxasulfone	+	6.51	383.1	203.0	-14.0	366.1	-9.0
63	Fenpyroximate	+	9.47	422.1	366.1	-16.0	135.0	-32.0
64	Fensulfothion	+	5.41	309.0	281.1	-15.0	253.1	-18.0
65	Fenthion (MPP)	+	3.97	279.1	264.0	-19.0	104.1	-28.0
	Fenthion oxon sulfone	+	4.05	295.1	78.1	-55.0	89.0	-52.0
	Fenthion oxon sulfoxide	+	3.98	279.0	264.0	-20.0	104.1	-28.0
	Fenthion oxon	+	5.71	263.0	231.0	-15.0	120.9	-45.0
	Fenthion sulfone	+	4.96	311.1	125.1	-21.0	109.1	-31.0
	Fenthion sulfoxide	+	4.85	295.1	109.0	-30.0	278.0	-20.0
66	Fentrazamide	+	7.41	350.2	83.1	-24.0	197.2	-8.0
67	Ferimzone *E*	+	5.81	255.1	132.1	-21.0	124.1	-22.0
	Ferimzone *Z*	+	5.81	255.1	132.1	-21.0	124.1	-22.0
68	Flonicamid	+	3.31	230.0	203.0	-17.0	174.0	-18.0
	TFNA	+	4.32	192.1	138.1	-29.0	165.0	-21.0
69	Florpyrauxifen-benzyl	+	7.91	439.0	65.0	-40.0	421.2	-17.0
70	Fluazinam	-	8.74	463.0	415.5	21.0	397.5	17.0
71	Flubendiamide	-	7.19	680.8	253.9	29.0	274.2	18.0
72	Fludioxonil	+	6.19	265.9	228.9	-15.0	157.9	-32.0
73	Flufenoxuron	+	9.19	489.0	158.1	-22.0	141.1	-47.0
74	Flumioxazine	+	5.70	355.3	327.1	-20.0	298.9	-30.0
75	Fluopicolide	+	6.34	382.8	173.0	-22.0	109.1	-55.0
76	Flupyrimin	+	4.74	316.0	90.0	-33.0	99.0	-39.0
77	Fluralaner	+	8.27	556.1	400.0	-27.0	160.1	-46.0
78	Fluridone	+	5.71	330.0	259.1	-49.0	294.1	-46.0
79	Flutolanil	+	7.02	341.1	87.0	-27.0	159.0	-10.0
80	Fluxametamide	+	8.91	474.1	399.9	-25.0	160.0	-36.0
81	Fluxapyroxad	+	6.29	382.1	362.1	-14.0	342.1	-21.0
82	Furametpyr	+	5.30	334.1	290.1	-27.0	131.1	-33.0
83	Hexaconazole	+	7.58	314.0	70.2	-23.0	159.0	-31.0
84	Imazalil	+	5.02	296.9	159.1	-24.0	69.2	-19.0
82	Inpyrfluxam	+	6.89	349.2	331.0	-23.0	315.1	-34.0
86	Ipfencarbazone	+	7.37	427.1	198.0	-14.0	128.0	-40.0
87	Ipflufenoquin	+	7.10	348.2	330.1	-13.0	314.1	-35.0
88	Iprobenfos	+	7.21	289.1	205.0	-15.0	65.1	-45.0
89	Isoprothiolane	+	6.35	291.0	231.0	-16.0	145.0	-28.0
90	Isoxathion	+	7.69	314.0	105.0	-10.0	286.0	-10.0
91	Lufenuron	-	8.85	508.9	326.0	22.0	175.0	36.0
92	Malathion	+	7.87	330.8	127.2	-13.0	125.1	-28.0
93	Mefenacet	+	6.53	299.0	120.2	-20.0	148.2	-22.0
94	Metaflumizone *E*	+	8.74	507.1	178.0	-25.0	287.2	-25.0
	Metaflumizone *Z*	+	8.74	507.1	178.0	-24.0	287.2	-26.0

95	Metalaxyl	+	5.41	280.1	160.1	-23.0	220.1	-24.0
96	Methamidophos	+	0.93	142.0	94.1	-15.0	125.1	-20.0
97	Methidathion	+	5.62	303.0	85.1	-22.0	145.0	-20.0
98	Methiocarb	+	6.06	226.1	121.1	-17.0	169.2	-10.0
99	Methoxyfenozide	+	6.42	369.2	149.1	-18.0	133.2	-23.0
100	Metominostrobin *E*	+	5.56	285.1	194.1	-15.0	166.1	-30.0
101	Metrafenone	+	7.82	408.9	209.2	-15.0	227.1	-21.0
102	Metyltetraprole	+	7.55	397.1	203.1	-25.0	91.1	-45.0
103	Molinate	+	6.49	188.0	126.2	-16.0	83.1	-19.0
104	Monocrotophos	+	3.51	224.0	127.0	-15.0	57.9	-26.0
105	Myclobutanil	+	7.21	289.1	205.0	-11.0	91.1	-10.0
106	Novaluron	+	8.31	492.8	158.1	-19.0	141.1	-45.0
107	Orysastrobin	+	6.34	392.2	116.1	-38.0	205.1	-25.0
108	Oxadiargyl	+	7.64	357.8	341.2	-11.0	223.0	-21.0
109	Oxathiapiprolin	+	6.35	540.2	500.1	-25.0	522.2	-27.0
110	Oxaziclomefone	+	8.41	376.1	133.1	-35.0	115.2	-43.0
111	Oxydemeton-methyl	+	3.28	246.9	169.0	-16.0	109.0	-30.0
112	Penconazole	+	7.21	284.2	159.0	-27.0	123.0	-50.0
113	Pencycuron	+	7.88	328.9	125.2	-10.0	89.0	-35.0
114	Pendimethalin	+	9.01	282.1	212.1	-12.0	134.1	-30.0
115	Penoxsulam	+	4.96	484.0	195.1	-27.0	164.1	-32.0
116	Phorate	+	3.96	260.9	75.1	-16.0	125.0	-16.0
	Phorate oxon sulfone	+	4.09	277.0	111.0	-24.0	127.0	-16.0
	Phorate oxon sulfoxide	+	3.98	261.0	111.1	-22.0	243.0	-9.0
	Phorate oxon	+	5.62	245.0	75.1	-11.0	47.0	-33.0
	Phorate sulfone	+	5.18	293.0	171.0	-11.0	143.0	-22.0
	Phorate sulfoxide	+	5.15	277.1	171.0	-14.0	142.9	-10.0
117	Phoxim	+	7.66	299.0	77.1	-29.0	129.2	-11.0
118	Pirimicarb	+	4.66	239.1	72.1	-28.0	182.2	-23.0
119	Probenazole	+	4.55	224.1	41.1	-14.0	196.0	-10.0
120	Profenofos	+	8.44	372.8	302.9	-19.0	128.0	-47.0
121	Propamocarb	+	4.31	189.8	163.0	-22.0	136.1	-10.0
122	Propiconazole	+	7.47	342.1	69.0	-22.0	41.1	-33.0
123	Propoxur	+	4.67	210.1	65.1	-34.0	168.1	-8.0
124	Propyrisulfuron	+	6.34	455.8	261.0	-18.0	218.1	-20.0
125	Prosulfocarb	+	8.29	252.1	91.2	-24.0	65.1	-55.0
126	Pydiflumetofen	+	7.82	426.1	406.1	-13.0	218.1	-20.0
127	Pyraclostrobin	+	7.65	388.1	149.0	-27.0	296.2	-15.0
128	Pyribencarb	+	5.80	362.1	207.0	-20.0	146.1	-24.0
129	Pyributicarb	+	8.89	331.2	190.1	-17.0	105.1	-36.0
130	Pyridaben	+	9.77	365.1	147.3	-15.0	309.2	-20.0
131	Pyriofenone	+	7.84	366.3	209.1	-25.0	166.1	-37.0

132	Pyriproxyfen	+	8.80	322.0	185.1	-22.0	227.1	-15.0
133	Pyroquilon	+	4.66	174.0	77.0	-43.0	117.1	-15.0
134	Quinoclamine	+	5.92	208.1	77.0	-36.0	95.1	-15.0
135	Saflufenacil	+	5.77	501.0	198.0	-44.0	349.0	-28.0
136	Sedaxane	+	6.51	332.2	292.1	-16.0	312.2	-14.0
137	Sethoxydim	+	8.61	328.1	178.2	-20.0	282.2	-11.0
138	Simazine	+	4.81	202.0	132.1	-17.0	96.2	-22.0
	Shimazine-2-hydroxy (OH-Simazine)	+	3.11	184.1	114.1	-18.0	69.1	-33.0
139	Spinetoram J	+	8.07	748.5	98.1	-54.0	115.3	-49.0
	Spinetoram L	+	8.48	760.3	142.2	-30.0	98.2	-55.0
140	Spinosyn A	+	7.59	732.2	142.2	-29.0	98.0	-55.0
	Spinosyn D	+	8.01	746.2	142.2	-29.0	98.0	-55.0
141	Spiromesifen	+	9.18	371.3	273.2	-16.0	255.2	-25.0
142	Spirotetramat	+	6.69	374.0	216.0	-34.0	302.1	-12.0
143	Sulfosulfuron	+	5.78	471.1	211.0	-14.0	218.0	-29.0
144	Sulfoxaflor	+	4.06	278.0	174.2	-10.0	154.1	-26.0
145	Tebuconazole	+	7.29	308.0	70.0	-22.0	125.0	-37.0
146	Tebufenozide	+	7.15	353.2	297.2	-8.0	105.1	-42.0
147	Tebufloquin	+	7.34	290.2	233.1	-34.0	218.1	-41.0
	Tebufloquin M1	+	6.00	248.2	218.2	-35.0	191.0	-37.0
148	Teflubenzuron	-	8.67	378.8	339.0	13.0	358.9	8.0
149	Terbuthylazine	+	6.23	230.2	174.1	-26.0	132.0	-24.0
	Terbuthylazine-2-hydroxy (OH-TER)	+	3.90	212.2	156.0	-16.0	86.2	-24.0
	Terbuthylazine-desethyl (DE-TER)	+	4.93	202.0	146.0	-16.0	79.1	-26.0
	Terbuthylazine-desethyl-2-hydroxy (OH-DE-TER)	+	2.86	184.1	128.1	-16.0	85.9	-24.0
150	Tetraniliprole	+	5.50	545.0	356.1	-14.0	375.9	-26.0
151	Thiabendazole	+	3.80	202.1	65.1	-46.0	77.1	-52.0
	5-Hydroxy thiabendazole	+	3.35	218.1	191.1	-25.0	81.0	-45.0
152	Thiacloprid	+	4.17	253.0	99.0	-43.0	90.1	-39.0
153	Thiamethoxam	+	3.39	292.0	211.1	-12.0	181.1	-22.0
154	Thiobencarb	+	7.79	258.0	125.2	-22.0	89.1	-49.0
155	Tiadinil	-	6.48	265.8	71.1	23.0	238.0	12.0
156	Tolprocarb	+	6.03	347.1	91.0	-30.0	65.0	-40.0
157	Trichlorfon (Metrifonate)	+	3.93	256.8	109.1	-20.0	221.0	-11.0
158	Tricyclazole	+	4.32	190.0	136.1	-26.0	163.1	-15.0
159	Trifloxystrobin	+	8.18	409.2	116.0	-23.0	131.1	-30.0
160	Triflumezopyrim	+	7.59	399.1	203.1	-15.0	146.1	-20.0

161	Warfarin	+	6.20	309.1	121.0	-41.0	147.1	-16.0

Supplementary material

Table S2. Coefficients of determination (R^2s) of calibration curves for all target compounds.

No.	Compound	Coefficients of determination (R^2)				
		Shrimp	Manila clam	Laver	Dried laver	Squid
1	Acephate	0.9984	0.9998	0.9993	0.9985	0.9825
2	Acetamiprid	0.9936	0.9931	0.9913	0.9971	0.9922
3	*N*-Desmethyl-acetamiprid(IM-2-1)	0.9913	0.9952	0.9939	0.9926	0.9886
4	Acynonapyr	0.9957	0.9814	0.9936	0.9987	0.9998
5	Alachlor	0.9979	0.9996	0.9981	0.9984	0.9905
6	Aldicarb	0.9982	0.9997	0.9999	0.9983	0.9952
7	Amitraz	0.9992	0.9994	0.9996	0.9998	0.9968
8	2,4-Dimethylaniline	0.9975	0.9989	0.9987	0.9996	0.9987
9	Atrazine	0.9896	0.9941	0.9964	0.9985	0.9935
10	Atrazine-desethyl (DEA)	0.9948	0.9964	0.9962	0.9942	0.9982
11	Atrazine-desisopropyl (DIA)	0.9978	0.9991	0.9981	0.9989	0.9992
12	Azinphos-methyl	0.9930	0.9962	0.9910	0.9913	0.9964
13	Azoxystrobin	0.9997	0.9996	0.9981	1.0000	0.9960
14	Bendiocarb	0.9964	0.9968	0.9955	0.9960	0.9988
15	Bensulfuron methyl	0.9960	0.9958	0.9975	0.9989	0.9998
16	Benzovindiflupyr	0.9895	0.9872	0.9933	0.9920	0.9862
17	Benzpyrimoxan	0.9956	0.9993	0.9985	0.9985	0.9845
18	Bifenazate	0.9971	0.9969	0.9995	0.9999	0.9880
19	Bifenazate-diazene	0.9957	0.9953	0.9989	0.9993	0.9968
20	Bioresmethrin	0.9999	0.9998	0.9998	0.9998	0.9998
21	Boscalid	0.9985	0.9987	0.9978	1.0000	0.9968
22	Brodifacoum	1.0000	0.9999	0.9999	0.9999	0.9997
23	Buprofezine	0.9971	0.9992	0.9968	0.9988	0.9990
24	Butamifos	0.9966	0.9977	0.9971	0.9956	0.9997
25	Cafenstrole	0.9851	0.9937	0.9837	0.9968	0.9812
26	Carbaryl	0.9989	0.9997	0.9998	0.9994	0.9984
27	Carbendazim	0.9997	0.9991	0.9994	0.9995	0.9977
28	Carbofuran	0.9987	0.9987	0.9982	0.9986	0.9998
29	3-Hydroxycarbofuran	0.9968	0.9989	0.9979	0.9961	0.9971
30	Carbosulfan	1.0000	1.0000	0.9997	0.9999	0.9997
31	Carfentrazone-ethyl	0.9973	0.9988	0.9981	0.9981	0.9925
32	Carpropamide	0.9977	0.9992	0.9967	0.9975	0.9957
33	Chlorantraniliprole	0.9952	0.9979	0.9968	0.9990	0.9889
34	Chlorfenvinphos *E*	0.9985	0.9979	0.9988	0.9998	0.9963
35	Chlorfenvinphos *Z*	0.9992	0.9991	0.9996	0.9984	0.9941
d36	Chromafenozide	0.9998	0.9995	0.9983	0.9991	0.9931
37	Clofentezine	0.9967	0.9997	0.9984	0.9958	0.9995
38	Clomeprop	0.9980	0.9997	0.9995	1.0000	0.9984
39	Clothianidin	0.9980	0.9983	0.9963	0.9952	0.9987
40	Cumyluron	0.9956	0.9955	0.9855	0.9949	0.9932
41	Cyantraniliprole	0.9957	0.9972	0.9994	0.9994	0.9990
42	Cyclopyrimorate	0.9985	0.9948	0.9978	0.9992	0.9823
43	Cyproconazole	0.9981	0.9991	0.9980	0.9982	0.9969
44	Daimuron	0.9940	0.9988	0.9955	0.9965	0.9908
45	Diazinon	0.9992	0.9989	0.9961	0.9978	0.9992
46	Dichlorvos (DDVP)	0.9987	0.9994	0.9989	0.9995	0.9980
47	Diclocymet *E*	0.9994	0.9987	0.9971	0.9973	0.9966
48	Diclocymet *Z*	0.9993	0.9988	0.9997	0.9987	0.9998
49	Diflubenzuron	0.9968	0.9970	0.9976	0.9958	0.9994
50	Dimethomorph *E*	0.9953	0.9934	0.9979	0.9992	0.9983
51	Dimethomorph *Z*	0.9994	0.9982	0.9966	0.9983	0.9974
52	Dinotefuran	0.9959	0.9931	0.9898	0.9980	0.9983
53	Disulfoton	0.9929	0.9933	0.9965	0.9959	0.9989
54	Disulfoton sulfone	0.9997	0.9989	0.9987	0.9985	0.9996
55	Disulfoton sulfoxide	0.9996	0.9989	0.9986	0.9996	0.9978
56	Demeton-S	0.9976	0.9992	0.9974	0.9975	0.9940
57	Demeton-S-sulfone	0.9961	0.9985	0.9942	0.9968	0.9982
58	Demeton-S-sulfoxide	0.9977	0.9984	0.9986	0.9961	0.9910

59	Diuron	0.9920	0.9953	0.9957	0.9981	0.9826
60	Edifenphos	0.9988	0.9992	0.9986	0.9993	0.9883
61	Emamectin benzoate	0.9982	0.9963	0.9993	1.0000	0.9996
62	Epoxyconazole	0.9988	0.9968	0.9991	0.9995	0.9939
63	Esprocarb	0.9970	0.9928	0.9972	0.9956	0.9901
64	Ethiofencarb	0.9994	0.9989	0.9989	0.9999	0.9972
65	Ethiprole	0.9861	0.9930	0.9825	0.9969	0.9976
66	Ethiprole-sulfone	0.9945	0.9983	0.9997	0.9996	0.9911
67	Ethoxyquin	0.9987	0.9994	0.9991	0.9896	0.9960
68	Ethoxyquin dimer	0.9974	0.9917	0.9966	0.9903	0.9997
69	Etobenzanid	0.9950	0.9967	0.9970	0.9973	0.9953
70	Etoxazole	0.9987	0.9997	0.9994	0.9965	0.9973
71	Etrimfos	0.9963	0.9986	0.9961	0.9968	0.9960
72	Famoxadone	0.9984	0.9973	0.9969	0.9986	0.9976
73	Fenamidone	0.9947	0.9954	0.9946	0.9983	0.9971
74	Fenarimol	0.9964	0.9927	0.9906	0.9961	0.9986
75	Fenbuconazole	0.9983	0.9919	0.9960	0.9983	0.9890
76	Fenhexamid	0.9981	0.9996	0.9982	0.9966	0.9994
77	Fenobucarb	0.9952	0.9968	0.9969	0.9994	0.9988
78	Fenoxasulfone	0.9829	0.9888	0.9870	0.9961	0.9808
79	Fenpyroximate	0.9994	0.9998	0.9999	0.9999	0.9990
80	Fensulfothion	0.9944	0.9958	0.9951	0.9983	0.9969
81	Fenthion(MPP)	0.9936	0.9979	0.9957	0.9965	0.9974
82	Fenthion oxon sulfone	0.9970	0.9959	0.9977	0.9984	0.9977
83	Fenthion oxon sulfoxide	0.9922	0.9962	0.9900	0.9975	0.9961
84	Fenthion oxon	0.9952	0.9981	0.9959	0.9955	0.9957
85	Fenthion sulfone	0.9974	0.9987	0.9986	0.9962	0.9982
86	Fenthion sulfoxide	0.9984	0.9984	0.9962	0.9982	0.9966
87	Fentrazamide	0.9889	0.9957	0.9850	0.9984	0.9943
88	Ferimzone *E*	0.9994	0.9997	0.9984	1.0000	0.9945
89	Ferimzone *Z*	0.9997	0.9997	0.9996	0.9999	0.9994
90	Flonicamid	0.9927	0.9957	0.9960	0.9970	0.9948
91	TFNA	0.9978	0.9983	0.9974	0.9986	0.9955
92	Florpyrauxifen-benzyl	0.9990	0.9993	0.9990	0.9998	0.9927
93	Fluazinam	0.9959	0.9883	0.9995	0.9994	0.9843
94	Flubendiamide	0.9909	0.9811	0.9857	0.9961	0.9912
95	Fludioxonil	0.9997	0.9998	0.9996	0.9992	0.9977
96	Flufenoxuron	0.9923	0.9958	0.9994	0.9984	0.9816
97	Flumioxazine	0.9919	0.9945	0.9949	0.9986	0.9877
98	Fluopicolide	0.9981	0.9984	0.9939	0.9973	0.9978
99	Flupyrimin	0.9992	0.9979	0.9878	0.9989	0.9984
100	Fluralaner	0.9963	0.9935	0.9974	0.9939	0.9912
101	Fluridone	0.9927	0.9985	0.9965	0.9986	0.9864
102	Flutolanil	0.9996	0.9972	0.9997	0.9981	0.9978
103	Fluxametamide	0.9953	0.9883	0.9934	0.9948	0.9906
104	Fluxapyroxad	0.9984	0.9996	0.9981	0.9989	0.9995
105	Furametpyr	0.9958	0.9952	0.9959	0.9978	0.9968
106	Hexaconazole	0.9993	0.9993	0.9999	0.9997	0.9975
107	Imazalil	0.9902	0.9921	0.9946	0.9998	0.9800
108	Inpyrfluxam	0.9975	0.9894	0.9946	0.9987	0.9929
109	Ipfencarbazone	0.9976	0.9988	0.9965	0.9991	0.9939
110	Ipflufenoquin	0.9954	0.9974	0.9984	0.9993	0.9970
111	Iprobenfos	0.9883	0.9959	0.9859	0.9993	0.9967
112	Isoprothiolane	0.9963	0.9988	0.9979	1.0000	0.9948
113	Isoxathion	0.9882	0.9997	0.9975	0.9990	0.9849
114	Lufenuron	0.9966	0.9997	0.9994	0.9989	0.9865
115	Malathion	0.9990	0.9966	0.9977	0.9981	0.9980
116	Mefenacet	0.9998	0.9997	0.9991	0.9998	0.9997
117	Metaflumizone *E*	0.9977	0.9897	0.9972	0.9998	0.9997
118	Metaflumizone *Z*	0.9925	0.9958	0.9989	0.9970	0.9946
119	Metalaxyl	0.9983	0.9997	0.9992	0.9995	0.9996
120	Methamidophos	0.9957	0.9937	0.9964	0.9978	0.9977
121	Methidathion	0.9954	0.9952	0.9971	0.9984	0.9936
122	Methiocarb	0.9991	0.9984	0.9973	0.9971	0.9990
123	Methoxyfenozide	0.9946	0.9989	0.9989	0.9985	0.9963
124	Metominostrobin *E*	0.9999	0.9988	0.9999	0.9997	0.9955

125	Metrafenone	0.9986	0.9945	0.9989	0.9987	0.9994
126	Metyltetraprole	0.9957	0.9976	0.9996	0.9995	0.9990
127	Molinate	0.9991	0.9992	0.9993	0.9999	0.9999
128	Monocrotophos	0.9868	0.9972	0.9837	0.9995	0.9845
129	Myclobutanil	0.9937	0.9931	0.9971	0.9996	0.9993
130	Novaluron	0.9999	0.9981	0.9998	0.9996	0.9991
131	Orysastrobin	0.9980	0.9986	0.9987	0.9999	0.9954
132	Oxadiargyl	0.9936	0.9978	0.9873	0.9975	0.9942
133	Oxathiapiprolin	0.9981	0.9988	0.9989	0.9998	0.9972
134	Oxaziclomefone	0.9992	0.9993	0.9995	0.9999	0.9980
135	Oxydemeton-methyl	0.9994	0.9996	0.9980	0.9997	0.9933
136	Penconazole	0.9983	0.9970	0.9961	0.9980	0.9973
137	Pencycuron	0.9999	0.9997	0.9993	1.0000	0.9996
138	Pendimethalin	0.9963	0.9977	0.9982	0.9962	0.9999
139	Penoxsulam	0.9990	0.9996	0.9992	0.9999	0.9964
140	Phorate	0.9992	0.9995	0.9959	0.9989	0.9957
141	Phorate oxon sulfone	0.9946	0.9970	0.9935	0.9971	0.9941
142	Phorate oxon sulfoxide	0.9980	0.9990	0.9984	0.9995	0.9985
143	Phorate oxon	0.9965	0.9957	0.9963	0.9957	0.9969
144	Phorate sulfone	0.9982	0.9982	0.9986	0.9997	0.9960
145	Phorate sulfoxide	0.9979	0.9978	0.9966	0.9990	0.9997
146	Phoxim	0.9898	0.9805	0.9959	0.9995	0.9984
147	Pirimicarb	0.9983	0.9981	0.9939	0.9995	0.9993
148	Probenazole	0.9984	0.9995	0.9922	0.9996	0.9985
149	Profenofos	0.9997	0.9975	0.9994	1.0000	0.9901
150	Propamocarb	0.9979	0.9967	0.9976	0.9997	0.9990
151	Propiconazole	0.9960	0.9964	0.9930	0.9988	0.9895
152	Propoxur	0.9888	0.9946	0.9953	0.9981	0.9996
153	Propyrisulfuron	0.9998	0.9993	0.9983	0.9999	0.9997
154	Prosulfocarb	0.9942	0.9936	0.9990	0.9992	0.9943
155	Pydiflumetofen	0.9992	0.9980	0.9984	0.9993	0.9961
156	Pyraclostrobin	0.9966	0.9970	0.9986	0.9970	0.9974
157	Pyribencarb	0.9920	0.9995	0.9990	0.9986	0.9978
158	Pyributicarb	0.9999	0.9999	0.9999	0.9999	0.9991
159	Pyridaben	0.9939	0.9961	0.9930	0.9993	0.9978
160	Pyriofenone	0.9996	0.9990	0.9994	0.9997	0.9902
161	Pyriproxyfen	0.9975	0.9979	0.9965	0.9999	0.9950
162	Pyroquilon	0.9985	0.9969	0.9984	0.9992	0.9977
163	Quinoclamine	0.9988	0.9961	0.9996	0.9999	0.9958
164	Saflufenacil	0.9974	0.9894	0.9943	0.9972	0.9802
165	Sedaxane	0.9988	0.9988	0.9998	0.9993	0.9972
166	Sethoxydim	0.9955	0.9971	0.9976	0.9982	0.9972
167	Simazine	0.9915	1.0000	0.9919	0.9940	0.9967
168	Shimazine-2-hydroxy (OH-Simazine)	0.9979	0.9984	0.9973	0.9998	0.9990
169	Spinetoram J	0.9988	0.9995	0.9996	1.0000	0.9995
170	Spinetoram L	0.9994	0.9978	0.9999	0.9997	0.9998
171	Spinosyn A	0.9996	0.9986	0.9999	0.9999	0.9993
172	Spinosyn D	0.9994	0.9987	0.9975	0.9986	0.9993
173	Spiromesifen	0.9971	0.9962	0.9984	0.9979	0.9914
174	Spirotetramat	0.9915	0.9917	0.9979	0.9929	0.9995
175	Sulfosulfuron	0.9963	0.9985	0.9970	0.9967	0.9958
176	Sulfoxaflor	0.9990	0.9987	0.9945	0.9981	0.9981
177	Tebuconazole	0.9977	0.9962	0.9991	0.9999	0.9956
178	Tebufenozide	0.9972	0.9993	0.9956	0.9987	0.9972
179	Tebufloquin	0.9976	0.9978	0.9982	0.9990	0.9919
180	Tebufloquin M1	0.9979	0.9992	0.9998	0.9997	0.9945
181	Teflubenzuron	0.9975	0.9982	0.9939	0.9989	0.9957
182	Terbuthylazine	0.9990	0.9986	0.9989	0.9988	0.9983
183	Terbuthylazine-2-hydroxy (OH-TER)	0.9921	0.9946	0.9934	0.9978	0.9948
184	Terbuthylazine-desethyl (DE-TER)	0.9991	1.0000	0.9963	0.9998	0.9946
185	Terbuthylazine-desethyl-2-hydroxy (OH-DE-TER)	0.9951	0.9968	0.9920	0.9994	0.9902
186	Tetraniliprole	0.9992	0.9991	0.9991	0.9996	0.9971
187	Thiabendazole	0.9981	0.9999	0.9990	0.9996	0.9983
188	5-Hydroxy thiabendazole	0.9991	0.9997	0.9995	0.9991	0.9952
189	Thiacloprid	0.9969	0.9970	0.9976	0.9996	0.9943
190	Thiamethoxam	0.9964	0.9995	0.9977	0.9982	0.9986

191	Thiobencarb	0.9990	0.9988	0.9980	0.9989	0.9983
192	Tiadinil	0.9981	0.9978	0.9995	0.9995	0.9959
193	Tolprocarb	0.9950	0.9991	0.9960	0.9977	0.9975
194	Trichlorfon (Metrifonate)	0.9957	0.9972	0.9953	0.9992	0.9969
195	Tricyclazole	0.9992	0.9998	0.9993	0.9992	0.9968
196	Trifloxystrobin	0.9977	0.9992	0.9979	0.9978	0.9910
197	Triflumezopyrim	0.9979	0.9992	0.9986	0.9985	0.9916
198	Warfarin	0.9935	0.9962	0.9934	0.9940	0.9955

Supplementary material

Table S3. Recoveries and coefficient of variations (CVs) of 161 pesticides and 37 of their metabolites at three concentrations using the method suggested by MFDS

		Compound	Conc.	Shrimp		Manila clam		Laver		Dried laver		Squid	
			(mg/kg)	Recovery (%)	CV (%)	Recovery (%)	CV (%)	Recovery (%)	CV (%)	Recovery (%)	CV (%)	Recovery (%)	CV (%)
1	1	Acephate	0.01	105.8	4.4	105.6	1.8	93.9	6.2	94.0	5.0	88.7	5.1
			0.02	104.3	3.6	101.6	1.9	105.3	4.0	94.5	3.6	99.1	16.8
			0.1	95.1	5.8	101.1	1.8	110.6	3.4	104.9	7.2	106.3	3.7
2	2	Acetamiprid	0.01	62.0	2.1	67.9	3.7	85.3	2.1	61.9	1.5	63.2	1.6
			0.02	99.0	2.2	100.7	1.4	112.0	4.1	94.0	1.6	95.1	7.3
			0.1	111.4	1.5	113.2	1.3	104.1	1.4	110.3	2.1	114.1	3.0
	3	*N*-Desmethyl-acetamiprid (IM-2-1)	0.01	62.4	3.0	72.3	4.7	89.4	1.3	65.1	2.6	62.5	0.9
			0.02	98.7	3.0	103.8	1.3	113.8	2.6	92.2	5.9	98.2	5.8
			0.1	113.6	1.4	111.4	2.0	102.7	1.8	118.2	1.4	111.4	2.6
3	4	Acynonapyr	0.01	118.6	1.3	83.3	9.0	107.4	0.8	118.0	2.6	-	-
			0.02	85.7	0.8	72.0	2.9	87.2	2.5	106.5	1.9	-	-
			0.1	77.3	2.4	91.5	9.8	96.7	1.5	110.7	1.6	-	-
4	5	Alachlor	0.01	85.5	4.9	100.3	7.5	91.8	5.1	77.7	4.9	72.6	14.8
			0.02	96.9	4.9	106.1	4.5	104.6	4.7	100.6	6.6	92.9	6.7
			0.1	109.8	1.4	110.2	2.2	103.7	3.1	106.7	2.8	107.5	4.9
5	6	Aldicarb	0.01	79.4	6.3	91.3	6.2	97.2	2.4	65.9	5.4	69.8	8.9
			0.02	97.6	2.9	97.0	3.3	102.7	6.9	88.0	0.7	97.0	4.6
			0.1	103.7	2.7	100.7	2.5	98.1	5.6	105.9	1.7	102.8	3.4
6	7	Amitraz	0.01	86.4	3.5	107.8	2.4	95.7	1.1	93.2	4.6	77.2	8.0
			0.02	99.6	2.0	103.1	1.6	104.6	4.1	99.8	3.4	100.6	4.7
			0.1	102.9	2.4	100.9	2.3	101.8	1.5	101.6	0.9	102.0	10.4
	8	2,4-Dimethylaniline	0.01	81.2	18.1	100.3	13.5	92.0	19.2	86.4	18.0	108.3	7.5
			0.02	90.9	9.4	94.0	10.9	92.6	11.1	88.1	6.5	107.3	6.6
			0.1	96.4	2.8	100.8	2.6	100.0	4.6	98.1	5.5	104.7	2.4
7	9	Atrazine	0.01	66.8	5.5	73.6	12.1	93.7	5.9	79.2	7.1	75.0	13.1
			0.02	95.6	11.1	94.2	7.1	104.9	8.3	91.2	4.1	101.2	6.2
			0.1	107.9	7.3	107.5	7.0	99.7	5.3	95.0	9.4	108.0	5.8
	10	Atrazine-desethyl (DEA)	0.01	66.7	7.4	75.0	5.4	91.2	7.5	76.3	4.0	75.8	8.6
			0.02	98.5	5.9	102.5	6.1	108.5	4.5	93.9	3.1	96.7	8.0
			0.1	110.5	1.3	111.6	1.3	103.1	2.7	105.3	2.3	104.6	3.6
	11	Atrazine-desisopropyl (DIA)	0.01	78.2	4.5	86.8	4.0	90.3	2.1	76.3	4.0	93.2	8.8
			0.02	96.0	2.5	99.6	2.0	104.2	3.4	93.9	3.1	103.2	3.9
			0.1	102.7	1.4	104.3	1.4	101.9	1.4	105.3	2.3	101.7	1.0
8	12	Azinphos-methyl	0.01	64.1	2.6	78.2	5.8	93.9	7.3	64.1	5.7	72.7	12.6
			0.02	98.2	8.3	109.6	6.5	116.0	4.1	86.0	13.6	101.8	5.6
			0.1	109.3	3.0	114.0	2.9	104.7	4.1	113.6	4.3	110.3	6.0
9	13	Azoxystrobin	0.01	91.0	4.1	100.1	5.2	95.4	1.5	97.2	2.9	112.1	3.8
			0.02	97.7	4.3	100.2	3.9	102.3	3.9	104.1	3.9	107.9	4.5
			0.1	103.9	3.1	101.5	7.5	101.4	4.9	101.0	6.9	104.8	2.3
10	14	Bendiocarb	0.01	73.1	4.9	85.4	6.6	90.6	4.1	61.2	2.1	86.0	13.7
			0.02	100.2	4.7	102.9	4.6	113.4	4.3	87.9	5.1	107.8	3.9

			0.1	107.0	4.0	110.9	2.9	107.7	2.5	111.6	2.9	106.7	2.8
11	15	Bensulfuron methyl	0.01	87.9	6.8	78.3	9.5	91.9	2.5	75.5	7.4	-	-
			0.02	89.6	7.4	91.1	6.5	101.9	9.5	94.3	5.3	-	-
			0.1	106.3	6.3	97.8	3.7	106.4	4.6	101.3	5.8	-	-
12	16	Benzovindiflupyr	0.01	62.7	3.4	69.2	9.5	82.9	15.1	62.3	1.4	68.8	9.1
			0.02	99.2	14.4	109.9	5.8	110.8	8.7	83.3	4.4	101.3	15.6
			0.1	117.8	3.6	116.3	4.2	108.3	5.3	111.5	5.2	116.1	4.5
13	17	Benzpyrimoxan	0.01	79.2	8.7	87.2	3.9	95.8	5.3	72.6	13.9	73.4	17.9
			0.02	93.2	1.9	98.0	5.7	112.8	3.2	96.6	8.0	89.5	6.4
			0.1	104.2	4.8	108.2	4.6	104.8	2.8	100.5	4.7	103.6	5.0
14	18	Bifenazate	0.01	82.3	8.8	83.3	7.4	96.5	11.9	91.8	10.7	72.3	14.4
			0.02	100.2	10.2	92.8	5.9	102.5	7.4	93.6	5.9	99.5	6.5
			0.1	104.1	9.3	104.6	3.5	105.3	9.1	92.9	10.4	110.8	6.2
	19	Bifenazate-diazene	0.01	81.8	7.1	76.3	10.0	95.2	5.0	-	-	76.6	10.5
			0.02	95.1	2.4	97.9	8.9	103.8	8.2	-	-	100.7	9.0
			0.1	97.7	4.1	107.7	3.0	105.5	7.8	-	-	113.7	6.2
15	20	Bioresmethrin	0.01	96.6	1.6	97.3	3.7	100.1	1.3	97.2	4.3	96.5	4.9
			0.02	104.1	2.2	101.5	0.9	103.3	3.0	102.9	5.8	98.1	2.6
			0.1	103.8	1.3	101.0	0.3	103.0	1.4	98.1	1.5	102.4	2.9
16	21	Boscalid	0.01	80.9	10.1	94.4	5.1	96.1	0.9	90.7	4.5	81.6	16.5
			0.02	100.5	3.8	103.1	5.6	101.7	2.5	95.1	8.6	96.5	9.6
			0.1	100.3	3.0	104.1	4.0	102.9	3.8	99.8	3.8	106.6	6.7
17	22	Brodifacoum	0.01	99.3	2.3	105.7	0.7	-	-	-	-	104.7	1.8
			0.02	102.5	1.6	101.5	0.7	-	-	-	-	103.9	1.5
			0.1	103.0	2.4	101.6	1.1	-	-	-	-	99.5	2.9
18	23	Buprofezine	0.01	78.0	5.0	89.3	2.3	89.4	4.4	72.6	4.1	101.8	11.5
			0.02	97.7	2.7	98.7	5.3	105.2	3.3	97.2	3.1	111.2	2.2
			0.1	106.8	3.6	104.7	0.8	103.9	1.5	105.8	2.9	104.7	8.0
19	24	Butamifos	0.01	83.3	2.9	80.5	5.6	89.9	7.4	65.2	3.8	98.0	10.0
			0.02	99.6	6.0	102.6	4.9	106.5	3.1	85.2	2.5	101.0	13.3
			0.1	106.5	2.3	102.3	4.8	101.8	2.6	101.7	2.2	103.3	7.0
20	25	Cafenstrole	0.01	67.0	10.9	69.7	12.3	81.3	8.4	66.3	7.3	67.2	8.8
			0.02	94.4	13.0	100.5	11.9	117.1	2.2	96.0	5.5	110.3	6.7
			0.1	114.7	3.0	106.0	4.7	109.4	7.9	101.8	6.8	110.2	8.8
21	26	Carbaryl	0.01	86.1	3.0	97.2	6.0	108.3	3.5	89.9	5.3	79.5	6.6
			0.02	98.3	5.1	102.7	3.4	106.6	4.1	101.4	5.4	97.6	5.2
			0.1	104.1	4.7	104.5	4.2	104.9	4.1	101.1	4.3	104.0	3.2
22	27	Carbendazim	0.01	95.8	2.6	86.8	7.2	108.8	2.3	80.2	5.2	86.0	5.5
			0.02	100.5	1.7	104.6	2.5	115.6	1.7	98.7	3.5	88.2	12.0
			0.1	101.4	0.4	99.7	4.7	106.1	16.0	107.0	1.3	104.1	3.8
23	28	Carbofuran	0.01	89.8	4.8	84.3	5.8	98.0	3.7	65.0	4.4	85.1	10.4
			0.02	100.3	5.7	96.8	3.1	107.7	5.7	85.6	3.8	104.7	8.8
			0.1	100.1	2.5	96.3	3.6	100.8	4.9	106.0	6.1	103.0	12.2
	29	3-Hydroxycarbofuran	0.01	67.5	9.0	86.6	11.9	95.0	4.1	63.7	6.1	73.0	9.6
			0.02	94.6	4.1	105.3	7.7	105.7	5.1	89.1	5.1	98.8	3.2
			0.1	106.6	1.8	109.3	1.9	101.8	1.8	104.4	1.6	106.4	1.3
24	30	Carbosulfan	0.01	-	-	100.3	1.7	95.8	1.6	96.8	1.6	90.0	5.3
			0.02	-	-	100.6	1.5	102.6	2.2	101.5	1.8	99.8	4.1
			0.1	-	-	101.1	1.4	97.5	2.4	101.5	1.1	109.3	2.4

25	31	Carfentrazone-ethyl	0.01	106.1	14.9	70.2	10.0	90.6	14.8	103.3	14.9	102.1	5.7
			0.02	109.2	10.0	86.4	13.0	113.9	3.5	102.7	10.3	93.5	19.3
			0.1	105.6	6.7	103.6	12.0	104.6	13.5	100.7	14.9	107.2	7.2
26	32	Carpropamide	0.01	83.5	5.1	92.8	3.8	88.7	3.8	65.4	6.7	79.1	14.2
			0.02	100.0	2.8	108.1	6.4	107.6	3.9	94.2	4.8	107.2	5.6
			0.1	111.7	4.6	107.5	4.6	105.5	2.4	109.1	2.2	108.1	6.9
27	33	Chlorantraniliprole	0.01	77.2	8.2	99.6	6.6	95.8	16.4	95.6	9.6	84.7	17.3
			0.02	100.7	8.9	105.3	9.1	104.0	8.8	106.8	8.0	89.8	13.7
			0.1	107.1	9.3	109.2	5.2	102.5	8.0	100.8	8.3	102.1	6.9
28	34	Chlorfenvinphos *E*	0.01	93.6	3.5	102.9	2.8	93.0	3.9	89.6	6.8	83.4	12.7
			0.02	100.9	3.4	104.1	5.6	103.8	2.8	102.3	4.6	101.2	12.1
			0.1	105.7	2.7	103.0	2.7	104.1	3.6	101.5	2.8	106.5	3.8
	35	Chlorfenvinphos *Z*	0.01	99.1	9.2	101.0	9.8	103.1	8.4	72.7	16.2	100.2	10.8
			0.02	104.3	5.9	103.3	7.5	106.6	4.5	85.9	2.6	97.4	11.3
			0.1	106.4	3.8	101.5	6.6	101.1	2.7	99.1	2.0	110.0	2.9
29	36	Chromafenozide	0.01	94.7	7.1	91.0	8.4	91.5	2.7	82.7	7.7	100.9	12.1
			0.02	100.3	6.3	94.8	6.3	105.6	6.5	92.9	10.8	104.9	7.5
			0.1	101.7	4.0	98.9	8.6	101.7	4.1	97.9	6.5	103.9	6.5
30	37	Clofentezine	0.01	71.4	14.7	91.5	6.8	93.4	6.5	71.4	9.4	98.4	10.8
			0.02	108.2	3.6	101.5	7.5	108.2	4.3	88.4	3.4	105.7	5.4
			0.1	109.7	6.8	102.0	6.9	102.7	4.1	106.4	3.7	97.3	8.1
31	38	Clomeprop	0.01	83.8	7.1	97.8	2.9	99.5	6.7	101.3	3.7	92.4	8.7
			0.02	92.3	3.8	101.5	6.8	98.7	2.8	103.0	3.6	104.8	3.4
			0.1	102.6	1.9	99.1	3.8	100.4	2.7	100.1	3.3	106.1	1.7
32	39	Clothianidin	0.01	79.6	3.9	88.9	5.6	92.4	3.4	62.7	2.4	82.8	6.2
			0.02	101.0	2.2	108.9	2.9	111.0	2.5	92.8	5.6	97.1	7.7
			0.1	100.7	3.1	110.1	1.9	107.5	2.9	105.4	6.7	103.4	4.4
33	40	Cumyluron	0.01	65.9	7.7	67.2	7.8	81.5	12.0	64.2	2.4	63.8	4.3
			0.02	96.9	13.5	98.8	9.0	106.5	6.3	92.2	6.7	100.3	15.6
			0.1	108.1	8.2	115.7	3.6	107.8	5.3	108.4	4.4	110.7	7.9
34	41	Cyantraniliprole	0.01	79.5	3.8	84.4	8.5	99.4	6.0	78.1	8.0	102.0	6.7
			0.02	101.8	7.1	98.1	7.7	105.2	6.4	98.5	9.9	97.6	7.9
			0.1	106.4	4.6	108.9	3.8	99.1	4.7	111.3	7.9	104.2	3.4
35	42	Cyclopyrimorate	0.01	87.5	5.3	67.6	13.2	86.0	7.5	-	-	63.0	3.5
			0.02	98.3	9.9	91.3	7.3	93.1	7.7	-	-	88.6	14.5
			0.1	105.5	5.4	99.0	8.0	100.2	7.8	-	-	102.0	7.4
36	43	Cyproconazole	0.01	84.9	12.6	85.0	4.8	92.6	5.6	64.2	1.7	73.1	16.0
			0.02	101.1	6.3	100.9	4.3	113.1	6.2	97.8	3.2	103.7	4.9
			0.1	103.5	3.4	107.3	3.1	102.3	6.3	111.5	4.3	103.6	3.2
37	44	Daimuron	0.01	76.7	17.3	66.8	10.7	85.2	10.7	76.6	13.3	62.1	5.1
			0.02	96.7	8.5	93.9	8.1	96.9	16.8	84.0	6.7	93.3	16.9
			0.1	104.7	7.7	113.8	4.8	96.6	8.4	103.7	6.6	111.3	6.6
38	45	Diazinon	0.01	94.0	3.9	107.2	7.0	89.7	6.1	63.3	3.0	84.7	10.6
			0.02	104.2	4.2	107.5	0.8	105.4	5.8	92.6	2.9	99.2	9.2
			0.1	104.2	4.0	101.3	5.9	104.7	5.4	107.9	4.2	109.9	8.3
39	46	Dichlorvos (DDVP)	0.01	87.4	4.6	96.2	5.6	105.0	2.4	78.0	2.2	89.7	6.8
			0.02	94.4	1.8	98.5	4.1	105.7	1.3	91.2	4.1	99.1	8.4
			0.1	100.2	1.8	99.3	2.9	104.1	1.5	101.0	2.6	101.9	4.3
40	47	Diclocymet *E*	0.01	107.5	6.8	65.4	8.9	79.8	17.6	63.7	5.0	95.2	11.2

			0.02	105.6	11.2	92.0	9.0	99.9	17.1	98.2	16.8	108.9	7.4
			0.1	109.8	3.5	105.7	3.5	97.0	4.3	107.8	2.8	103.1	6.0
	48	Diclocymet *Z*	0.01	82.8	17.1	67.4	10.1	99.6	4.1	80.3	17.0	85.1	10.8
			0.02	101.2	15.6	87.3	5.8	103.4	8.4	94.9	10.4	99.2	10.2
			0.1	112.9	6.3	101.4	5.4	103.1	6.9	101.4	6.5	106.4	5.8
41	49	Diflubenzuron	0.01	91.9	11.9	110.0	7.8	93.3	11.0	70.0	10.8	86.5	19.6
			0.02	104.1	6.5	111.0	6.3	106.4	5.5	90.6	11.0	98.8	15.2
			0.1	105.0	7.1	110.3	4.3	104.3	6.7	107.8	4.4	95.1	8.8
42	50	Dimethomorph *E*	0.01	75.9	14.0	75.2	6.8	92.4	7.8	73.6	5.1	88.1	8.3
			0.02	97.7	9.6	101.0	8.0	104.4	6.5	94.5	3.8	95.9	12.2
			0.1	107.5	3.9	108.4	5.6	99.3	6.3	104.3	7.0	106.5	9.5
	51	Dimethomorph *Z*	0.01	63.1	3.5	67.1	9.9	91.4	3.2	62.1	2.4	100.0	14.4
			0.02	105.2	11.9	104.3	6.5	110.3	8.0	90.1	7.0	112.7	3.2
			0.1	112.4	7.0	104.8	4.2	101.9	5.7	100.1	4.7	102.7	9.9
43	52	Dinotefuran	0.01	94.9	4.5	86.9	1.5	85.5	11.9	89.1	6.7	94.5	5.0
			0.02	96.3	6.6	95.1	5.8	106.5	3.0	90.5	4.6	100.8	4.0
			0.1	101.1	2.8	101.1	4.3	107.8	5.7	100.2	6.1	105.7	3.1
44	53	Disulfoton	0.01	93.2	1.4	91.2	3.9	92.3	5.7	74.7	6.2	85.3	4.6
			0.02	103.5	1.3	96.5	3.0	107.4	2.8	99.2	6.9	96.9	5.5
			0.1	104.5	2.9	102.6	0.7	105.2	1.5	107.7	2.3	105.5	6.8
	54	Disulfoton sulfone	0.01	74.5	6.4	91.7	4.2	99.0	2.7	67.6	8.8	69.8	11.7
			0.02	101.9	1.5	104.5	2.9	112.0	4.4	95.5	5.4	99.7	8.3
			0.1	107.3	5.5	109.4	2.4	105.4	0.5	106.3	3.5	104.2	2.5
	55	Disulfoton sulfoxide	0.01	78.5	2.4	102.7	2.7	89.1	4.7	63.3	2.5	87.6	4.7
			0.02	101.7	3.0	105.3	0.7	108.1	2.5	91.1	4.3	103.7	6.7
			0.1	109.4	3.0	110.8	3.4	103.9	3.2	110.6	2.2	105.4	6.4
	56	Demeton-S	0.01	63.5	3.9	87.7	3.9	93.7	4.8	62.2	2.7	64.9	8.9
			0.02	100.2	9.4	104.5	6.9	107.4	4.4	92.6	3.1	101.9	9.6
			0.1	108.7	5.4	107.5	2.6	102.4	2.2	110.3	2.9	111.6	6.0
	57	Demeton-S-sulfone	0.01	85.1	4.3	91.5	4.5	87.4	4.5	85.0	4.8	85.7	10.0
			0.02	102.5	2.6	103.6	1.1	106.5	4.7	104.4	5.2	97.4	7.2
			0.1	105.0	1.5	108.2	3.4	107.0	1.4	107.2	2.3	104.7	5.6
	58	Demeton-S-sulfoxide	0.01	88.3	4.9	94.5	2.7	95.9	2.8	102.2	3.5	85.7	6.9
			0.02	98.4	1.3	100.7	1.7	101.8	2.9	100.9	3.1	104.4	6.7
			0.1	103.6	2.5	106.7	1.6	104.7	1.1	97.6	4.1	108.1	1.5
45	59	Diuron	0.01	79.3	10.1	110.5	4.8	101.6	4.1	107.8	5.7	66.7	9.8
			0.02	100.8	5.6	112.1	2.9	107.7	5.8	109.8	4.9	98.6	3.4
			0.1	105.7	2.4	107.6	2.8	105.8	3.4	102.3	4.7	112.8	2.8
46	60	Edifenphos	0.01	84.1	9.9	88.5	2.7	92.8	6.4	62.3	2.5	78.6	9.6
			0.02	100.0	6.2	102.1	5.3	107.4	3.2	87.2	6.4	97.8	11.9
			0.1	107.9	4.8	107.5	4.7	101.1	4.0	102.3	7.1	110.5	8.0
47	61	Emamectin benzoate	0.01	88.5	1.2	103.5	1.6	95.6	2.7	102.2	3.5	91.7	9.2
			0.02	95.5	1.3	100.7	3.1	101.5	2.4	100.9	3.1	100.1	7.1
			0.1	104.2	1.5	102.8	3.0	103.2	1.1	97.6	4.1	108.0	3.3
48	62	Epoxyconazole	0.01	97.7	10.4	112.2	5.8	94.7	1.5	107.8	5.7	82.6	14.4
			0.02	97.7	5.7	105.0	3.8	97.2	10.2	109.8	4.9	101.1	7.8
			0.1	102.5	3.8	101.6	7.2	100.4	6.7	102.3	4.7	98.4	12.5
49	63	Esprocarb	0.01	87.7	13.8	80.0	6.5	93.5	13.5	62.3	2.5	66.5	8.8
			0.02	101.5	6.2	97.7	6.6	106.7	4.5	87.2	6.4	101.5	8.1

			0.1	105.3	4.5	107.6	5.7	101.5	2.5	102.3	7.1	104.3	8.5
50	64	Ethiofencarb	0.01	91.0	5.0	85.2	5.0	98.3	5.3	109.6	1.8	63.7	4.3
			0.02	96.2	5.3	89.0	8.7	94.9	5.7	99.6	8.3	88.6	3.2
			0.1	98.4	5.1	96.7	4.7	96.9	10.0	94.2	4.3	101.9	2.4
51	65	Ethiprole	0.01	63.3	4.6	64.6	4.2	84.3	12.7	65.8	3.0	89.1	19.4
			0.02	99.5	5.9	101.2	15.5	111.4	5.3	90.2	7.6	111.9	6.8
			0.1	105.1	10.1	112.3	6.6	105.7	10.9	111.8	5.4	113.4	7.2
	66	Ethiprole-sulfone	0.01	94.0	16.3	114.7	7.4	94.2	15.3	107.7	5.6	98.1	14.4
			0.02	94.0	18.3	94.6	13.3	107.9	12.1	106.8	8.4	106.6	8.5
			0.1	97.8	4.8	88.1	7.4	104.8	10.4	96.5	6.0	106.6	10.3
52	67	Ethoxyquin	0.01	89.6	10.9	89.1	11.4	105.5	5.1	-	-	88.1	8.5
			0.02	107.3	8.5	100.2	12.5	106.0	10.1	-	-	106.6	5.9
			0.1	108.3	7.2	105.5	6.8	100.4	2.9	-	-	99.0	6.1
	68	Ethoxyquin dimer	0.01	99.1	4.5	100.0	19.9	-	-	85.7	17.4	96.1	4.1
			0.02	103.4	5.1	94.4	16.0	-	-	92.3	10.0	101.6	1.5
			0.1	106.6	4.2	113.2	8.7	-	-	100.7	18.6	104.0	1.2
53	69	Etobenzanid	0.01	71.0	18.0	81.4	14.4	102.3	4.5	72.0	10.6	73.7	18.0
			0.02	106.8	9.4	103.6	8.4	112.9	5.8	90.2	6.3	98.0	12.6
			0.1	111.1	4.8	107.1	3.7	101.8	7.6	104.8	5.8	104.7	9.7
54	70	Etoxazole	0.01	97.6	3.3	99.9	3.0	95.1	7.1	68.0	3.2	99.7	10.7
			0.02	99.4	3.1	104.0	7.4	103.9	3.4	86.9	6.9	103.8	4.9
			0.1	104.6	6.2	106.1	6.3	101.8	6.8	101.2	3.7	104.1	6.9
55	71	Etrimfos	0.01	70.8	6.7	95.8	5.6	93.0	6.3	62.3	3.6	78.5	16.7
			0.02	93.5	2.2	104.3	6.2	106.2	7.3	89.8	3.7	100.1	9.5
			0.1	108.8	4.0	105.1	4.0	102.6	2.7	105.8	1.8	106.9	5.5
56	72	Famoxadone	0.01	80.8	10.0	94.7	13.7	91.7	10.6	70.1	12.9	99.0	5.9
			0.02	98.6	9.6	105.0	11.9	103.0	5.6	92.9	4.4	108.7	4.0
			0.1	102.9	4.2	105.8	2.8	100.7	5.1	108.0	4.4	104.1	4.7
57	73	Fenamidone	0.01	73.7	11.0	74.5	10.1	80.8	4.3	64.4	6.4	101.8	4.4
			0.02	104.8	6.5	98.5	5.2	115.9	3.4	93.8	2.7	102.2	8.0
			0.1	111.7	5.2	111.0	5.1	106.3	3.2	105.5	4.6	104.8	5.1
58	74	Fenarimol	0.01	93.6	10.8	82.0	18.9	93.9	6.9	64.8	9.6	84.6	9.0
			0.02	112.5	7.0	94.2	19.6	101.0	12.3	95.0	8.3	99.5	7.0
			0.1	108.3	5.6	107.4	11.3	107.3	7.9	106.2	6.5	103.7	3.8
59	75	Fenbuconazole	0.01	97.8	10.6	95.9	9.0	88.2	5.7	69.4	11.1	96.6	18.0
			0.02	104.9	7.1	103.1	2.9	104.4	2.9	91.9	12.3	103.9	11.9
			0.1	101.1	3.1	109.4	3.5	109.6	1.9	103.1	5.2	104.5	5.6
60	76	Fenhexamid	0.01	79.4	7.5	101.1	4.6	95.7	5.0	62.8	6.2	83.2	8.3
			0.02	95.6	3.5	106.1	1.8	107.9	4.4	89.3	10.4	98.8	4.5
			0.1	104.0	6.9	107.4	5.4	100.1	5.5	107.3	3.9	102.4	5.5
61	77	Fenobucarb	0.01	81.0	7.3	73.2	16.8	92.0	7.1	96.5	4.2	80.0	14.0
			0.02	95.5	4.1	91.4	5.4	99.6	8.2	99.2	6.8	92.1	16.6
			0.1	103.2	3.6	97.3	5.4	100.4	8.0	101.2	7.1	104.4	6.5
62	78	Fenoxasulfone	0.01	65.4	5.5	68.4	12.7	91.5	14.3	63.4	3.4	64.3	6.6
			0.02	111.1	5.5	110.9	2.8	115.7	2.4	99.6	4.2	92.2	15.7
			0.1	109.5	6.0	112.7	4.6	110.0	7.0	101.2	3.6	101.1	6.4
63	79	Fenpyroximate	0.01	93.4	3.4	104.4	2.0	98.5	1.8	97.0	2.8	91.5	4.3
			0.02	96.2	1.2	100.9	1.7	100.9	2.1	96.9	1.6	98.0	3.9
			0.1	103.3	1.4	99.5	2.0	101.5	2.3	100.4	1.4	104.8	5.4

64	80	Fensulfothion	0.01	64.4	5.2	71.8	8.0	90.5	5.0	64.3	5.6	93.4	7.3
			0.02	97.9	6.0	103.5	3.4	112.3	3.3	96.3	6.2	99.9	9.1
			0.1	113.2	3.5	110.3	1.9	103.8	3.6	105.5	3.6	106.3	4.3
65	81	Fenthion (MPP)	0.01	62.9	3.1	83.1	3.1	87.8	1.6	60.7	1.2	72.1	12.0
			0.02	93.3	2.6	104.0	3.6	107.2	1.1	85.5	1.3	98.7	5.1
			0.1	107.0	3.1	109.2	0.7	101.8	2.9	106.5	3.1	107.4	4.0
	82	Fenthion oxon sulfone	0.01	67.9	10.2	80.7	9.8	84.1	14.4	64.1	2.5	70.9	11.7
			0.02	103.0	5.5	106.3	6.1	107.4	2.2	94.0	4.9	93.3	11.8
			0.1	108.7	5.2	114.8	4.9	105.1	1.8	102.5	2.6	107.9	7.1
	83	Fenthion oxon	0.01	68.7	3.4	82.2	2.3	92.1	1.4	61.6	2.0	81.9	8.6
		sulfoxide	0.02	97.6	6.6	105.8	1.1	107.7	2.2	91.5	5.2	100.2	3.0
			0.1	106.4	3.2	109.6	3.0	101.5	1.7	111.4	3.0	103.2	6.6
	84	Fenthion oxon	0.01	81.0	9.8	78.6	6.9	94.6	7.8	75.7	7.5	70.8	8.2
			0.02	103.3	6.4	98.1	1.9	107.1	5.9	101.2	8.4	95.3	12.2
			0.1	108.6	5.2	104.3	7.1	106.4	7.9	110.3	3.9	106.6	7.5
	85	Fenthion sulfone	0.01	72.1	15.6	103.4	13.0	91.1	13.0	68.1	4.6	70.9	11.7
			0.02	104.6	5.7	107.7	6.3	108.0	5.0	91.7	7.5	93.3	11.8
			0.1	111.2	2.6	111.6	2.8	109.3	2.3	108.9	4.4	107.9	7.1
	86	Fenthion sulfoxide	0.01	80.6	7.3	94.2	7.9	84.8	3.9	68.1	4.6	81.9	8.6
			0.02	98.2	6.3	103.9	4.3	106.5	4.7	91.7	7.5	100.2	3.0
			0.1	109.0	4.0	111.1	0.8	103.8	3.8	108.9	4.4	103.2	6.6
66	87	Fentrazamide	0.01	67.3	11.3	76.6	13.3	82.9	6.4	68.5	8.4	70.1	8.8
			0.02	93.2	8.4	101.4	3.4	113.1	4.3	91.4	10.3	92.5	4.4
			0.1	106.6	8.3	109.5	6.5	104.1	4.0	108.8	1.1	113.7	4.7
67	88	Ferimzone *E*	0.01	91.8	2.7	93.6	3.2	93.1	4.6	102.2	4.5	69.3	6.5
			0.02	94.5	4.2	95.7	3.9	100.6	2.7	102.6	4.2	92.0	15.8
			0.1	98.7	2.6	98.5	2.1	102.0	3.9	101.8	2.4	108.5	4.6
	89	Ferimzone *Z*	0.01	101.8	1.8	97.5	4.5	95.4	2.2	92.3	4.6	89.5	5.4
			0.02	98.8	3.5	97.3	1.2	97.8	3.4	97.1	3.6	97.4	2.3
			0.1	99.2	1.2	98.8	1.4	100.7	2.6	100.5	2.6	101.5	5.0
68	90	Flonicamid	0.01	63.2	3.1	103.8	10.1	94.1	18.1	75.7	12.1	77.4	15.5
			0.02	76.9	4.5	99.6	13.5	108.5	6.1	95.3	7.0	92.4	14.4
			0.1	100.5	3.2	110.3	4.3	101.9	2.0	111.8	3.9	102.1	6.7
	91	TFNA	0.01	88.7	6.3	85.0	4.0	92.6	3.9	81.9	6.6	72.6	11.2
			0.02	100.1	5.3	91.4	8.5	104.0	5.6	100.7	7.6	92.2	5.2
			0.1	105.3	2.2	99.8	2.2	100.0	3.9	102.4	6.0	111.5	4.0
69	92	Florpyrauxifen-benzyl	0.01	98.6	9.0	83.4	17.3	101.0	9.4	69.3	9.6	72.7	8.7
			0.02	105.9	7.9	101.5	4.6	106.5	7.7	88.0	11.5	98.3	0.9
			0.1	108.5	5.4	105.3	5.3	101.0	5.9	98.1	4.0	108.2	7.8
70	93	Fluazinam	0.01	91.2	4.6	97.7	5.9	88.7	7.0	86.5	7.1	68.9	11.8
			0.02	99.0	2.2	95.5	3.3	100.3	4.9	99.0	5.8	100.2	11.8
			0.1	102.7	2.7	106.3	3.4	100.3	5.1	100.4	3.9	98.6	7.2
71	94	Flubendiamide	0.01	71.7	16.9	83.1	10.1	77.8	14.0	99.4	11.1	80.9	16.5
			0.02	112.8	4.3	97.6	11.2	99.4	16.4	95.0	12.6	97.4	19.4
			0.1	109.2	5.1	101.8	8.2	103.0	5.2	91.1	7.7	107.8	10.0
72	95	Fludioxonil	0.01	64.1	6.3	65.8	9.4	92.0	12.0	63.9	4.7	70.0	10.8
			0.02	99.1	3.7	103.5	4.6	106.8	5.0	92.8	4.7	91.3	8.7
			0.1	115.5	3.0	117.0	3.1	105.8	4.9	109.5	3.7	108.0	1.4
73	96	Flufenoxuron	0.01	92.3	2.2	104.8	3.2	94.6	3.1	81.2	4.5	75.8	8.3

			0.02	96.3	3.2	104.1	1.9	104.8	4.9	92.8	5.6	95.9	10.4
			0.1	103.7	3.4	105.2	1.9	105.8	2.5	101.6	1.8	105.3	3.0
74	97	Flumioxazine	0.01	82.0	10.6	78.4	14.5	103.0	6.3	111.8	5.0	85.6	17.4
			0.02	100.5	19.7	105.3	16.5	110.3	8.7	104.2	18.2	98.9	18.9
			0.1	102.8	11.1	112.7	8.5	99.7	7.7	110.7	5.3	92.4	9.3
75	98	Fluopicolide	0.01	64.8	4.0	71.5	10.2	90.0	6.8	68.3	11.4	68.3	11.2
			0.02	111.8	4.0	109.9	9.1	113.8	5.2	102.1	4.5	101.7	8.6
			0.1	118.0	1.2	113.3	5.0	101.5	4.5	106.3	1.8	113.0	3.6
76	99	Flupyrimin	0.01	86.2	8.2	91.7	9.9	93.2	8.4	61.3	1.3	82.1	16.4
			0.02	97.8	8.0	110.2	5.2	104.1	4.7	88.9	6.1	96.9	7.9
			0.1	109.3	5.7	113.0	5.6	104.7	5.7	99.1	3.1	101.4	8.7
77	100	Fluralaner	0.01	105.0	11.1	98.1	8.7	101.9	6.5	116.7	3.1	74.3	15.2
			0.02	101.5	7.8	104.1	5.0	100.6	5.6	103.0	3.8	91.0	7.5
			0.1	97.7	8.6	99.0	14.7	108.8	8.1	92.5	10.2	100.6	8.2
78	101	Fluridone	0.01	72.4	9.9	67.2	8.0	89.4	9.0	64.1	4.0	69.8	4.9
			0.02	103.0	9.6	103.0	8.3	106.8	10.9	90.4	7.7	93.2	12.7
			0.1	101.6	7.4	117.3	1.7	106.0	8.5	111.2	5.8	105.4	9.5
79	102	Flutolanil	0.01	77.7	5.8	95.1	11.7	89.4	12.1	70.8	12.9	76.0	11.5
			0.02	93.4	9.2	109.8	6.2	104.3	7.1	97.3	5.1	93.5	14.8
			0.1	106.3	4.0	109.2	3.9	103.2	7.7	107.1	3.3	101.0	7.6
80	103	Fluxametamide	0.01	101.9	11.3	114.9	4.7	98.9	6.5	117.4	1.5	90.4	13.3
			0.02	99.6	10.1	99.0	8.8	93.9	6.4	107.8	5.8	90.9	18.9
			0.1	104.8	7.4	100.1	8.2	108.8	4.1	91.6	4.6	93.4	6.9
81	104	Fluxapyroxad	0.01	65.6	8.4	67.5	5.2	105.3	12.5	61.7	2.2	70.9	4.4
			0.02	97.9	11.9	102.1	8.9	115.3	5.4	107.5	9.8	102.6	13.7
			0.1	108.7	6.9	113.8	9.8	98.5	6.9	108.8	4.6	115.0	4.9
82	105	Furametpyr	0.01	78.2	6.4	98.6	0.8	98.6	4.1	77.9	4.4	108.6	6.6
			0.02	98.4	3.1	102.1	6.5	106.0	2.8	99.1	1.8	106.6	5.3
			0.1	101.3	4.6	108.2	3.5	104.1	3.9	105.6	1.7	99.9	3.5
83	106	Hexaconazole	0.01	65.9	8.0	77.3	10.7	91.4	7.9	64.4	2.0	85.3	13.6
			0.02	98.4	3.9	99.9	3.5	105.5	5.4	90.9	7.5	98.8	5.4
			0.1	106.9	3.9	105.2	2.9	104.5	2.8	105.2	4.6	105.8	4.1
84	107	Imazalil	0.01	97.5	1.6	104.1	2.1	100.5	2.6	87.2	6.1	87.3	4.2
			0.02	101.1	1.8	104.1	3.3	100.5	3.2	103.6	4.6	99.6	8.0
			0.1	100.7	3.7	102.4	0.8	100.9	4.1	100.2	2.1	103.1	6.7
85	108	Inpyrfluxam	0.01	75.5	13.2	111.2	7.9	87.9	6.5	82.1	14.1	102.2	16.7
			0.02	95.3	8.9	117.0	2.5	106.6	10.8	102.3	8.0	106.4	9.3
			0.1	106.4	6.8	108.5	5.2	104.0	7.0	106.9	5.8	101.9	10.9
86	109	Ipfencarbazone	0.01	74.4	11.8	64.1	8.8	87.8	11.2	70.6	13.1	81.3	18.0
			0.02	108.1	7.6	106.1	5.1	112.9	5.6	102.4	9.2	99.9	14.9
			0.1	109.8	7.7	108.1	10.0	106.3	11.4	108.2	3.2	115.1	2.6
87	110	Ipflufenoquin	0.01	82.6	9.5	90.8	5.2	96.8	14.0	65.5	7.4	73.5	17.9
			0.02	95.2	7.6	102.9	5.4	100.9	4.9	95.6	4.9	90.7	13.1
			0.1	105.1	7.2	104.4	4.1	108.1	6.1	104.0	8.9	94.6	13.8
88	111	Iprobenfos	0.01	78.4	9.4	104.6	7.2	99.9	4.7	80.2	10.9	103.4	4.7
			0.02	95.4	5.7	103.8	5.1	108.3	7.3	97.9	2.2	112.0	5.7
			0.1	107.0	7.6	104.2	7.3	105.3	6.8	99.0	6.1	105.0	6.2
89	112	Isoprothiolane	0.01	63.5	5.3	73.0	12.5	87.4	11.1	85.9	4.6	69.9	9.2
			0.02	99.6	4.1	97.5	8.5	104.7	6.1	97.0	4.2	104.2	7.6

			0.1	112.2	3.7	108.2	6.4	105.1	10.6	107.3	4.2	114.4	7.6
90	113	Isoxathion	0.01	86.0	6.0	103.6	5.3	93.0	7.2	96.1	6.5	73.2	8.4
			0.02	96.9	7.7	99.5	3.7	103.4	6.4	101.6	3.9	96.2	7.4
			0.1	105.1	6.2	100.7	7.1	100.5	3.6	102.6	4.9	104.6	10.1
91	114	Lufenuron	0.01	107.5	8.7	102.6	8.8	108.9	5.5	117.3	1.8	68.7	7.6
			0.02	106.1	4.7	97.8	9.9	96.8	4.4	105.2	4.1	99.4	9.9
			0.1	100.3	5.2	94.8	4.5	105.1	4.7	96.8	4.5	116.6	2.2
92	115	Malathion	0.01	78.5	6.9	90.9	7.5	103.7	3.3	83.6	15.4	74.4	12.1
			0.02	99.8	2.0	104.8	7.2	101.9	5.4	105.4	3.2	99.9	10.5
			0.1	108.3	7.3	107.9	3.8	104.0	4.7	110.3	4.0	112.0	7.3
93	116	Mefenacet	0.01	96.3	7.9	89.6	5.3	92.4	4.3	66.1	4.4	98.0	13.4
			0.02	107.9	7.0	104.2	5.6	100.2	5.9	94.1	6.8	95.7	6.1
			0.1	104.8	3.6	108.3	4.3	103.7	3.0	102.0	6.3	101.2	4.6
94	117	Metaflumizone *E*	0.01	104.1	4.5	102.4	13.4	96.5	6.6	83.4	13.5	108.7	5.6
			0.02	103.4	3.5	98.7	11.5	100.7	6.2	95.0	5.5	110.3	3.9
			0.1	104.1	2.5	102.7	2.3	100.9	2.2	101.9	4.8	103.2	3.5
	118	Metaflumizone *Z*	0.01	93.1	10.3	114.1	4.6	100.8	13.7	111.0	10.7	80.4	9.5
			0.02	94.9	18.5	106.6	6.8	99.7	9.0	114.1	4.7	97.4	9.4
			0.1	105.3	4.4	106.1	11.3	105.7	5.6	103.1	10.5	104.7	6.9
95	119	Metalaxyl	0.01	74.3	5.6	81.7	4.4	93.9	10.1	61.8	1.8	71.8	15.7
			0.02	93.9	4.5	95.0	5.5	104.7	4.6	92.5	6.8	92.3	4.0
			0.1	109.5	3.0	106.3	5.6	108.0	3.9	105.4	1.8	109.2	6.5
96	120	Methamidophos	0.01	83.8	11.0	108.6	2.3	101.3	5.6	94.6	8.4	94.3	5.1
			0.02	90.5	7.2	107.0	4.7	112.2	3.2	107.2	1.3	103.4	4.1
			0.1	103.3	6.2	105.7	3.4	104.7	3.9	101.6	4.7	95.7	7.1
97	121	Methidathion	0.01	77.6	13.1	77.1	9.7	81.9	8.5	65.0	2.9	74.4	10.3
			0.02	98.6	7.5	102.8	5.4	105.3	3.8	99.6	4.2	107.9	4.8
			0.1	111.1	1.0	109.4	2.6	103.5	3.8	105.1	5.6	113.3	4.4
98	122	Methiocarb	0.01	73.0	10.8	92.2	9.0	92.6	5.1	64.3	5.5	76.0	11.4
			0.02	95.0	10.1	102.4	8.5	106.4	3.4	91.9	9.2	90.7	5.6
			0.1	107.1	5.3	103.3	4.0	99.8	8.0	103.3	9.0	104.6	3.9
99	123	Methoxyfenozide	0.01	83.3	8.9	102.1	2.1	94.0	9.0	62.6	1.3	76.5	14.6
			0.02	103.3	4.4	105.6	5.9	106.8	1.6	94.0	5.6	109.3	9.8
			0.1	109.6	2.7	109.1	1.8	102.3	3.8	108.1	1.9	110.7	5.4
100	124	Metominostrobin *E*	0.01	97.4	2.7	101.5	3.5	98.0	3.7	109.1	4.1	75.9	3.1
			0.02	96.8	3.1	102.3	3.9	102.6	3.8	102.4	4.2	95.7	6.1
			0.1	100.7	3.9	104.9	2.4	102.6	5.0	97.4	3.5	103.9	8.3
101	125	Metrafenone	0.01	105.3	2.3	106.4	4.1	93.7	6.9	72.3	10.8	100.9	4.4
			0.02	105.2	5.3	103.8	3.7	101.6	3.5	92.3	6.1	107.3	5.1
			0.1	109.2	2.8	100.6	3.6	102.5	1.0	106.2	4.0	99.0	10.1
102	126	Metyltetraprole	0.01	69.3	12.4	90.1	2.1	93.4	6.6	65.4	6.0	90.1	12.5
			0.02	98.5	7.4	96.0	6.5	105.3	4.1	91.3	10.6	102.7	12.2
			0.1	106.7	3.4	102.2	5.4	102.8	1.6	103.5	5.1	114.7	4.3
103	127	Molinate	0.01	92.6	15.8	94.1	17.7	94.0	10.5	107.0	11.3	90.9	5.6
			0.02	98.3	11.9	94.5	16.9	103.3	11.1	102.3	13.0	102.7	5.3
			0.1	99.3	6.3	104.2	6.4	100.1	9.9	100.4	4.6	100.5	4.6
104	128	Monocrotophos	0.01	94.5	15.7	113.2	7.0	100.2	6.7	112.2	9.4	108.4	10.6
			0.02	103.1	15.9	109.4	8.3	94.9	11.3	107.4	5.6	105.5	6.0
			0.1	103.0	6.9	101.0	6.0	100.5	5.8	102.8	5.9	99.0	3.6

105	129	Myclobutanil	0.01	63.9	2.4	85.7	7.7	73.4	10.8	75.8	10.2	71.9	17.4
			0.02	86.3	2.0	97.5	4.5	106.2	7.7	100.7	5.4	90.4	11.6
			0.1	115.9	2.7	108.8	8.3	109.8	2.9	103.2	6.9	106.6	11.0
106	130	Novaluron	0.01	84.2	5.8	91.0	11.6	96.1	12.4	92.3	15.6	107.7	8.2
			0.02	97.1	12.3	107.3	12.0	110.4	7.7	112.2	7.0	101.4	8.5
			0.1	102.6	3.9	94.2	3.8	105.9	5.2	107.9	4.7	93.4	6.7
107	131	Orysastrobin	0.01	99.4	2.7	103.2	7.3	98.0	4.9	84.4	1.8	112.0	6.8
			0.02	105.7	5.0	103.7	5.3	102.3	5.0	95.0	6.1	113.8	5.3
			0.1	101.8	4.5	99.3	3.4	103.2	4.7	101.4	5.6	106.9	6.1
108	132	Oxadiargyl	0.01	83.0	18.5	101.9	13.6	94.4	7.8	92.3	18.9	74.5	7.7
			0.02	101.0	10.3	106.8	7.9	96.3	10.1	108.3	5.8	105.4	8.0
			0.1	103.3	6.2	106.4	7.0	102.6	5.1	112.3	1.9	112.8	5.1
109	133	Oxathiapiprolin	0.01	65.6	8.6	87.1	6.0	83.1	6.7	62.1	2.3	69.8	9.1
			0.02	98.7	7.0	111.9	2.4	111.4	6.3	100.7	3.7	97.9	4.0
			0.1	111.1	1.6	106.6	6.0	107.3	5.2	108.3	2.8	114.3	6.1
110	134	Oxaziclomefone	0.01	87.8	8.2	107.5	7.7	98.3	6.9	89.6	9.3	82.0	17.5
			0.02	107.8	5.2	105.7	3.3	105.9	3.8	103.9	10.4	92.9	10.7
			0.1	110.8	6.1	104.0	2.6	103.7	11.1	101.7	8.7	101.5	11.0
111	135	Oxydemeton-methyl	0.01	106.4	3.9	107.3	4.6	96.8	2.7	97.9	2.9	82.2	3.8
			0.02	107.0	6.0	103.0	3.4	99.2	3.6	100.1	1.6	95.0	2.9
			0.1	100.0	4.0	99.2	3.0	101.8	1.9	102.8	2.1	106.0	1.7
112	136	Penconazole	0.01	89.1	2.6	99.7	7.7	90.6	5.6	91.6	6.0	76.9	9.1
			0.02	99.1	3.2	108.7	3.6	107.6	5.7	100.4	3.5	95.2	7.7
			0.1	104.4	5.4	105.0	3.0	104.0	4.9	100.1	2.0	103.8	8.2
113	137	Pencycuron	0.01	84.9	6.3	82.4	3.9	95.9	3.4	62.6	3.0	84.3	12.4
			0.02	99.7	4.4	102.1	7.7	99.4	5.9	92.2	6.8	96.8	8.0
			0.1	106.0	7.7	107.3	2.7	101.1	4.2	106.3	5.1	106.4	7.1
114	138	Pendimethalin	0.01	100.2	4.3	103.1	7.5	99.6	4.5	101.6	3.8	88.1	5.5
			0.02	95.0	5.6	103.5	2.7	101.8	6.6	97.3	8.4	97.8	8.6
			0.1	97.1	2.8	100.4	4.6	100.7	1.8	94.8	4.6	105.2	4.3
115	139	Penoxsulam	0.01	75.9	13.5	110.1	4.4	90.5	10.2	-	-	-	-
			0.02	98.8	13.6	105.8	6.2	108.7	6.1	-	-	-	-
			0.1	104.9	4.0	110.3	6.1	98.6	6.9	-	-	-	-
116	140	Phorate	0.01	86.6	5.2	98.0	4.6	96.2	6.6	101.3	3.8	77.2	16.4
			0.02	98.1	2.7	104.1	2.6	103.3	3.8	100.7	1.9	99.9	5.9
			0.1	102.6	2.7	103.3	1.4	103.3	3.5	102.1	3.4	104.7	6.4
	141	Phorate oxon sulfone	0.01	67.5	6.0	80.2	4.3	86.2	3.2	63.5	2.7	69.5	6.1
			0.02	97.2	3.3	104.2	5.6	108.1	3.4	94.0	2.6	97.5	7.8
			0.1	105.8	3.5	114.8	2.8	99.1	3.8	110.7	3.5	110.0	2.8
	142	Phorate oxon sulfoxide	0.01	81.9	2.2	94.4	3.0	89.7	2.6	78.9	2.4	93.1	11.6
			0.02	96.7	3.5	104.9	3.2	101.7	3.8	96.3	1.7	99.6	4.3
			0.1	104.9	2.1	106.1	1.3	101.4	1.9	103.3	1.5	100.5	6.6
	143	Phorate oxon	0.01	84.6	7.4	109.2	3.4	93.8	8.6	90.9	7.3	70.9	6.7
			0.02	102.2	3.7	111.1	4.5	101.6	6.7	95.1	3.1	97.6	6.0
			0.1	108.4	4.3	104.5	6.2	100.6	3.1	106.0	5.5	108.5	8.4
	144	Phorate sulfone	0.01	90.4	6.1	92.1	11.0	98.0	8.6	63.0	2.0	68.0	9.5
			0.02	93.0	6.3	108.2	7.5	105.5	4.6	89.8	10.7	100.7	6.3
			0.1	105.9	4.4	104.4	5.6	99.5	3.0	104.2	6.6	108.2	7.1
	145	Phorate sulfoxide	0.01	76.4	4.7	82.4	6.4	91.7	6.5	93.3	3.6	68.1	10.9

			0.02	93.4	3.8	98.1	4.7	104.4	2.2	101.9	2.9	96.0	7.1
			0.1	106.8	2.0	103.2	3.9	103.4	1.2	101.8	2.4	106.3	3.5
117	146	Phoxim	0.01	73.3	6.4	93.2	7.1	96.6	6.8	71.9	11.1	100.1	3.2
			0.02	97.4	7.0	105.5	3.4	112.8	4.6	94.0	7.1	107.6	2.1
			0.1	107.4	4.3	105.1	5.0	103.8	3.6	101.3	4.7	104.2	3.6
118	147	Pirimicarb	0.01	62.4	3.9	62.4	2.2	93.6	3.9	112.3	3.4	104.1	8.2
			0.02	81.6	2.6	83.9	7.2	101.6	4.2	102.0	4.9	98.9	3.7
			0.1	106.3	4.5	111.2	4.6	107.6	4.8	98.4	5.3	102.3	8.0
119	148	Probenazole	0.01	78.1	16.1	89.0	12.2	93.0	9.2	-	-	-	-
			0.02	99.8	5.7	107.8	6.7	111.8	5.8	-	-	-	-
			0.1	107.8	3.9	115.6	2.0	110.0	4.2	-	-	-	-
120	149	Profenofos	0.01	102.3	8.1	101.5	2.8	82.5	8.7	82.8	12.6	88.0	11.5
			0.02	103.8	11.7	109.0	8.2	105.7	4.9	96.9	5.9	101.7	10.4
			0.1	109.5	1.9	99.1	5.4	97.3	9.8	101.6	10.4	106.8	8.6
121	150	Propamocarb	0.01	93.8	2.0	82.5	6.6	100.2	1.5	105.2	6.7	82.5	12.7
			0.02	99.0	4.3	94.3	7.1	99.3	5.8	113.4	4.4	93.6	3.1
			0.1	105.6	1.3	105.2	4.0	103.4	5.0	115.4	2.0	106.6	7.5
122	151	Propiconazole	0.01	86.1	6.8	85.4	10.8	93.6	5.0	85.8	14.1	86.8	16.1
			0.02	97.9	2.0	101.6	4.0	103.7	1.9	94.7	7.7	101.4	4.4
			0.1	102.8	2.2	106.5	3.4	104.5	3.8	98.2	4.8	104.0	7.5
123	152	Propoxur	0.01	71.4	8.4	83.5	9.2	85.4	3.7	70.9	10.2	68.1	9.2
			0.02	100.9	4.5	96.5	4.1	110.8	6.1	99.0	6.3	95.5	14.2
			0.1	110.1	3.7	111.0	3.8	106.9	4.5	112.1	5.8	104.6	8.4
124	153	Propyrisulfuron	0.01	65.5	5.1	76.4	6.2	89.8	7.2	-	-	-	-
			0.02	102.3	9.9	102.1	9.5	112.4	6.0	-	-	-	-
			0.1	116.5	2.4	109.8	3.8	101.7	3.5	-	-	-	-
125	154	Prosulfocarb	0.01	94.4	6.2	103.5	2.7	91.7	6.5	100.8	4.8	89.7	5.9
			0.02	102.1	8.4	102.2	3.3	100.3	5.2	98.2	2.2	98.6	4.3
			0.1	103.1	4.5	100.2	6.1	99.2	3.2	96.3	3.6	109.1	7.0
126	155	Pydiflumetofen	0.01	82.0	14.3	79.1	16.2	92.4	15.9	65.0	5.4	78.3	15.4
			0.02	92.4	7.5	103.8	7.2	103.3	12.7	102.7	5.8	99.8	15.6
			0.1	109.5	8.5	113.5	4.5	95.8	10.2	97.8	6.8	111.5	9.0
127	156	Pyraclostrobin	0.01	95.5	6.0	101.0	6.1	93.0	6.5	90.4	1.3	81.2	14.9
			0.02	103.6	6.2	104.3	3.8	103.0	3.4	96.4	7.1	103.1	10.7
			0.1	102.9	4.3	108.5	2.8	100.9	3.8	102.2	3.9	115.2	8.0
128	157	Pyribencarb	0.01	72.3	8.6	90.9	7.1	98.7	6.5	68.2	14.0	94.3	10.4
			0.02	100.0	4.0	104.9	4.5	109.4	8.3	89.1	6.6	102.3	7.8
			0.1	115.0	3.3	106.8	7.1	103.0	3.9	107.8	2.8	97.2	7.3
129	158	Pyributicarb	0.01	86.9	3.1	109.7	2.9	100.1	9.7	75.2	12.9	92.4	8.3
			0.02	94.5	9.2	103.2	3.0	108.1	3.7	97.7	5.4	100.4	5.2
			0.1	104.4	4.3	101.0	4.2	102.8	2.8	111.5	5.1	105.8	9.2
130	159	Pyridaben	0.01	96.4	3.0	103.8	0.7	97.0	1.3	99.1	1.2	96.5	3.4
			0.02	100.2	1.3	100.6	1.7	100.8	2.4	97.6	2.1	103.1	5.6
			0.1	102.1	1.4	101.2	0.8	102.3	2.8	96.8	0.8	105.7	1.7
131	160	Pyriofenone	0.01	76.9	4.4	91.6	8.3	80.4	9.6	70.3	6.4	79.1	14.3
			0.02	98.5	2.8	108.1	6.0	102.6	7.3	98.8	6.5	100.6	15.2
			0.1	104.2	3.5	110.6	4.2	103.7	4.9	104.7	8.9	106.0	7.4
132	161	Pyriproxyfen	0.01	91.8	3.4	95.7	2.6	97.2	5.8	87.3	6.1	66.7	5.7
			0.02	100.3	2.8	101.2	4.6	102.1	4.6	97.9	4.2	82.4	7.5

			0.1	103.5	1.8	106.4	3.4	100.9	1.9	100.1	2.5	102.4	6.4
133	162	Pyroquilon	0.01	81.6	9.6	99.1	5.8	91.5	10.6	98.6	6.0	82.5	17.0
			0.02	98.5	4.8	102.3	5.4	98.2	4.2	104.5	5.7	98.5	11.0
			0.1	101.0	5.0	104.5	2.5	99.1	5.6	102.6	2.0	110.3	7.1
134	163	Quinoclamine	0.01	94.1	4.8	84.1	5.9	108.4	6.1	109.1	7.1	78.1	14.3
			0.02	99.0	2.9	91.3	6.2	103.8	7.8	106.8	8.0	86.8	11.7
			0.1	101.0	7.1	99.2	6.4	103.3	6.2	98.5	9.5	113.3	4.1
135	164	Saflufenacil	0.01	99.8	12.6	79.6	10.5	85.1	6.8	-	-	78.8	9.7
			0.02	93.8	12.0	90.7	12.5	103.5	4.5	-	-	95.1	7.6
			0.1	103.5	5.0	101.8	2.5	107.0	5.3	-	-	109.4	4.7
136	165	Sedaxane	0.01	80.9	18.1	64.1	6.8	89.0	10.3	63.4	18.3	75.0	10.9
			0.02	97.7	15.4	94.6	9.9	110.0	7.2	92.5	12.9	88.2	11.1
			0.1	106.8	5.6	113.2	3.6	104.7	4.8	102.7	7.5	110.2	10.7
137	166	Sethoxydim	0.01	97.0	2.4	91.9	3.4	96.9	3.0	-	-	87.7	8.4
			0.02	101.4	3.7	97.3	5.2	100.9	3.1	-	-	97.5	10.5
			0.1	101.4	2.7	105.5	2.0	102.6	3.7	-	-	107.3	7.2
138	167	Simazine	0.01	75.4	8.1	89.2	11.3	98.5	6.7	64.5	8.0	78.6	19.3
			0.02	95.7	7.5	97.5	3.5	109.1	6.6	89.9	4.3	99.6	15.3
			0.1	106.5	4.0	110.4	1.8	102.6	3.9	109.2	3.1	118.4	1.1
	168	Shimazine-2-hydroxy	0.01	-	-	-	-	-	-	-	-	-	-
		(OH-Simazine)	0.02	-	-	-	-	-	-	-	-	-	-
			0.1	-	-	-	-	-	-	-	-	-	-
139	169	Spinetoram J	0.01	84.0	13.5	95.5	12.0	115.7	2.5	-	-	84.5	15.7
			0.02	92.9	5.1	102.4	9.6	103.8	8.6	-	-	88.2	17.5
			0.1	102.1	3.2	106.3	4.1	104.2	6.6	-	-	105.5	7.5
	170	Spinetoram L	0.01	110.2	2.2	105.0	4.0	99.2	2.2	-	-	96.6	6.3
			0.02	99.5	2.1	101.8	2.4	100.7	4.3	-	-	105.6	9.9
			0.1	100.3	2.5	104.4	2.5	101.7	2.2	-	-	102.4	4.3
140	171	Spinosyn A	0.01	89.4	2.8	103.6	3.2	100.1	1.4	-	-	98.0	6.5
			0.02	97.3	2.8	102.8	5.4	100.7	3.6	-	-	98.7	10.7
			0.1	107.4	2.5	103.6	2.2	101.3	1.9	-	-	105.4	6.5
	172	Spinosyn D	0.01	99.3	5.6	102.2	2.5	103.5	3.2	-	-	99.4	5.8
			0.02	99.3	3.8	99.3	2.4	103.3	2.9	-	-	97.9	11.1
			0.1	104.3	4.4	104.5	2.3	100.8	3.8	-	-	100.5	1.8
141	173	Spiromesifen	0.01	87.0	14.9	109.3	6.8	96.7	6.4	85.3	4.1	101.1	12.3
			0.02	109.5	6.3	107.5	6.6	99.9	10.5	98.9	5.3	104.2	11.9
			0.1	107.6	4.1	113.3	4.8	101.1	5.8	99.7	4.6	107.2	5.2
142	174	Spirotetramat	0.01	97.2	6.4	102.2	3.2	93.1	8.7	75.1	8.3	92.4	17.0
			0.02	98.0	0.9	100.2	3.3	105.6	8.0	90.6	8.8	106.4	6.4
			0.1	101.0	3.0	102.0	3.0	101.3	4.1	105.6	3.0	108.5	6.6
143	175	Sulfosulfuro	0.01	76.5	5.2	71.6	11.5	103.4	6.7	-	-	83.5	18.3
			0.02	95.2	5.4	93.4	7.5	111.9	9.7	-	-	101.8	11.9
			0.1	105.6	11.3	109.0	3.2	106.6	2.9	-	-	107.6	7.6
144	176	Sulfoxaflor	0.01	77.3	6.0	87.1	5.5	89.9	1.8	61.3	1.9	71.9	5.7
			0.02	100.3	5.8	100.0	3.7	108.5	1.6	93.2	5.2	101.4	6.5
			0.1	109.4	0.8	108.9	1.7	104.4	2.0	108.5	3.1	108.6	4.2
145	177	Tebuconazole	0.01	90.9	8.2	99.4	5.2	101.1	4.7	64.0	5.3	79.9	12.3
			0.02	102.6	4.0	102.6	10.1	108.8	5.7	89.8	11.3	92.0	8.6
			0.1	95.4	3.8	103.2	2.8	108.0	8.3	105.7	4.9	110.3	5.1

146	178	Tebufenozide	0.01	93.8	3.3	108.6	5.6	93.8	3.9	99.0	11.3	82.5	11.4
			0.02	103.0	7.3	104.9	4.7	102.7	4.2	107.6	7.0	105.3	7.2
			0.1	101.0	7.6	101.2	6.7	102.3	6.4	94.3	12.3	104.6	6.4
147	179	Tebufloquin	0.01	87.5	11.1	88.7	5.8	86.8	5.7	62.7	3.8	95.0	15.1
			0.02	94.9	6.7	103.1	2.6	107.9	2.3	90.5	7.2	105.5	8.3
			0.1	106.9	2.6	108.7	2.6	104.4	3.8	102.1	5.3	105.5	7.5
	180	Tebufloquin M1	0.01	89.5	11.8	98.9	6.8	96.9	6.1	69.1	8.7	81.4	15.1
			0.02	102.2	6.1	105.5	6.5	108.5	2.7	102.3	4.8	102.8	11.2
			0.1	105.6	6.3	108.2	7.8	103.8	5.3	100.0	6.8	110.7	7.8
148	181	Teflubenzuron	0.01	87.3	9.7	112.2	9.5	96.5	4.9	108.6	9.3	70.8	6.3
			0.02	102.1	9.3	106.3	7.6	101.5	5.3	103.8	5.6	99.3	3.5
			0.1	103.4	3.4	99.1	5.8	100.0	5.6	93.9	4.1	111.8	2.5
149	182	Terbuthylazine	0.01	95.7	3.5	83.0	13.4	87.6	9.0	72.7	16.0	67.3	13.1
			0.02	95.8	9.5	100.3	12.9	98.8	8.5	90.4	5.5	102.4	15.8
			0.1	108.6	3.0	101.1	2.2	105.4	5.8	107.5	4.1	105.2	9.1
	183	Terbuthylazine-2-	0.01	-	-	-	-	-	-	-	-	-	-
		hydroxy (OH-TER)	0.02	-	-	-	-	-	-	-	-	-	-
			0.1	-	-	-	-	-	-	-	-	-	-
	184	Terbuthylazine-	0.01	64.1	2.6	79.0	4.7	86.7	6.1	62.2	1.9	72.0	10.7
		desethyl (DE-TER)	0.02	95.2	3.3	104.8	2.9	112.5	2.3	89.9	6.5	94.6	10.1
			0.1	112.5	4.2	109.8	3.8	102.3	1.4	105.7	2.7	105.3	4.3
	185	Terbuthylazine-	0.01	-	-	-	-	-	-	-	-	-	-
		desethyl-2-	0.02	-	-	-	-	-	-	-	-	-	-
		hydroxy(OH-DE-TER)	0.1	-	-	-	-	-	-	-	-	-	-
150	186	Tetraniliprole	0.01	89.9	9.7	94.0	11.3	91.7	10.6	82.5	10.4	79.0	15.6
			0.02	97.5	18.5	101.6	9.5	100.1	6.7	100.0	3.1	87.4	18.6
			0.1	101.5	8.3	104.1	5.8	103.8	8.1	108.7	2.6	92.9	10.4
151	187	Thiabendazole	0.01	113.4	1.7	103.0	3.4	101.7	3.3	83.2	4.4	105.6	3.9
			0.02	117.3	1.1	100.4	0.5	93.8	14.0	91.8	4.6	101.8	1.6
			0.1	94.0	4.0	103.7	1.1	107.0	3.8	100.3	4.4	101.0	4.7
	188	5-Hydroxy	0.01	86.6	1.9	99.3	1.5	91.9	2.7	82.0	6.3	90.1	6.3
		thiabendazole	0.02	95.1	1.2	103.2	4.1	99.9	5.1	97.0	5.3	105.4	8.2
			0.1	99.8	3.7	101.3	3.1	102.2	2.9	101.8	2.7	108.6	2.1
152	189	Thiacloprid	0.01	76.9	4.2	89.3	4.7	92.9	5.8	82.0	6.3	83.8	9.8
			0.02	100.5	4.4	99.6	2.4	108.1	4.7	97.0	5.3	102.9	6.2
			0.1	106.6	2.8	109.2	5.5	106.9	1.7	101.8	2.7	109.2	5.4
153	190	Thiamethoxam	0.01	97.4	10.9	92.0	1.1	92.2	2.0	62.1	2.2	74.4	2.1
			0.02	106.5	5.3	103.5	1.1	105.7	1.9	95.0	4.4	97.6	4.7
			0.1	109.8	1.2	106.1	1.8	104.1	2.0	113.2	1.5	109.4	2.3
154	191	Thiobencarb	0.01	91.2	3.4	100.1	2.3	93.0	2.7	74.4	8.8	74.6	5.3
			0.02	104.5	4.5	104.4	4.9	109.5	3.8	94.8	3.3	97.1	4.1
			0.1	106.1	3.8	105.2	5.2	102.9	4.0	106.6	2.1	107.1	2.3
155	192	Tiadinil	0.01	116.8	1.8	101.4	5.6	102.9	11.1	109.4	6.7	81.1	15.3
			0.02	102.5	4.6	94.1	6.4	95.9	6.7	99.8	6.4	91.1	8.1
			0.1	101.3	5.2	98.6	3.2	99.2	6.1	102.5	4.3	112.4	4.7
156	193	Tolprocarb	0.01	74.1	6.9	88.2	6.0	88.3	5.8	64.6	4.4	75.3	10.4
			0.02	97.1	3.2	103.2	3.8	110.5	5.1	93.3	4.0	104.1	8.3
			0.1	106.2	3.4	109.8	6.0	101.3	2.6	101.1	5.1	108.9	6.2

157	194	Trichlorfon (Metrifonate)	0.01	94.6	10.1	95.1	16.2	109.7	10.7	73.9	6.9	113.8	4.9
			0.02	87.3	6.8	105.9	6.0	116.8	2.4	92.9	9.3	81.3	6.5
			0.1	107.9	1.9	111.0	1.6	109.5	1.1	107.6	4.8	111.4	2.1
158	195	Tricyclazole	0.01	88.1	3.6	98.1	2.7	96.1	3.5	71.7	7.3	82.3	4.7
			0.02	101.8	1.2	101.4	1.0	104.3	3.9	94.0	3.4	95.1	5.4
			0.1	104.5	1.6	104.8	2.4	103.0	1.3	103.8	2.0	107.4	3.3
159	196	Trifloxystrobin	0.01	90.8	6.1	100.2	4.0	92.4	4.6	65.6	6.5	88.4	7.1
			0.02	98.5	4.9	102.5	3.7	97.3	3.0	89.3	5.7	106.9	6.8
			0.1	102.6	5.1	106.4	4.3	108.1	3.1	105.9	4.0	104.4	10.2
160	197	Triflumezopyrim	0.01	84.9	9.2	89.1	6.4	95.3	5.3	70.6	10.2	94.5	11.1
			0.02	100.6	4.0	98.7	5.7	106.5	4.8	95.6	5.1	106.1	11.0
			0.1	102.3	3.5	103.4	4.4	106.7	3.4	100.1	2.3	102.0	9.4
161	198	Warfarin	0.01	66.8	8.3	78.7	9.2	86.0	5.0	-	-	73.7	11.4
			0.02	98.7	8.6	106.3	4.0	112.9	3.1	-	-	98.0	10.1
			0.1	112.4	5.0	109.4	4.3	111.0	3.8	-	-	107.9	7.0

Supplementary material

Table S4. Matrix effects on the 198 compounds in shrimp, manila clam, laver, dried laver and squid

		Compound	Matrix effect (%) (n=1)				
			Shrimp	Manila clam	Laver	Dried laver	Squid
1	1	Acephate	8.7	17.4	8.1	26.2	1.8
2	2	Acetamiprid	0.8	8.9	5.0	9.4	8.2
	3	*N*-Desmethyl-acetamiprid (IM-2-1)	11.5	19.4	8.5	22.9	2.4
3	4	Acynonapyr	470.1	20059.1	393.1	14501.3	2065.8
4	5	Alachlor	0.8	15.3	19.1	11.4	13.6
5	6	Aldicarb	1.0	5.4	11.4	3.8	1.8
6	7	Amitraz	0.3	20.2	23.9	40.3	14.6
	8	2,4-Dimethylaniline	2.8	25.3	13.7	20.1	0.0
7	9	Atrazine	1.4	12.3	12.8	10.8	0.8
	10	Atrazine-desethyl (DEA)	1.4	2.5	11.2	21.5	7.5
	11	Atrazine-desisopropyl (DIA)	0.0	33.1	10.4	15.8	53.5
8	12	Azinphos-methyl	3.1	7.8	1.1	1.2	9.2
9	13	Azoxystrobin	7.5	3.7	12.7	1.8	1.1
10	14	Bendiocarb	0.1	18.6	5.5	10.8	0.3
11	15	Bensulfuron methyl	8.4	17.4	12.5	7.3	8.7
12	16	Benzovindiflupyr	18.2	16.9	14.7	0.9	16.0
13	17	Benzpyrimoxan	6.8	4.1	11.5	10.2	3.9
14	18	Bifenazate	20.6	11.3	15.1	7.5	4.1
	19	Bifenazate-diazene	6.6	8.4	27.2	2.1	26.3
15	20	Bioresmethrin	6.5	37.0	10.4	50.4	16.5
16	21	Boscalid	9.3	5.2	18.2	9.8	11.5
17	22	Brodifacoum	5.6	24.0	14.1	52.1	7.0
18	23	Buprofezine	27.8	14.0	21.9	7.8	5.5
19	24	Butamifos	4.9	2.9	0.7	12.1	1.7
20	25	Cafenstrole	0.5	16.8	35.4	5.5	14.3
21	26	Carbaryl	2.9	10.3	6.2	12.2	2.1
22	27	Carbendazim	11.1	10.5	3.2	4.7	11.2
23	28	Carbofuran	5.5	14.3	12.0	7.2	15.4
	29	3-Hydroxycarbofuran	4.5	16.3	1.1	12.4	0.4

24	30	Carbosulfan	1.2	70.6	7.8	22.2	69.2
25	31	Carfentrazone-ethyl	2.7	5.6	20.9	1.9	19.1
26	32	Carpropamide	7.2	7.3	8.7	3.8	10.2
27	33	Chlorantraniliprole	8.1	11.8	22.9	2.4	6.8
28	34	Chlorfenvinphos *E*	12.1	4.1	1.5	0.7	2.2
	35	Chlorfenvinphos *Z*	0.8	5.4	5.4	29.5	2.5
29	36	Chromafenozide	8.0	6.7	10.2	24.3	2.7
30	37	Clofentezine	12.0	17.7	8.8	30.7	4.8
31	38	Clomeprop	6.8	11.2	2.3	27.3	8.0
32	39	Clothianidin	6.1	7.6	3.5	1.1	9.5
33	40	Cumyluron	18.7	17.3	11.3	8.6	0.6
34	41	Cyantraniliprole	8.0	9.8	10.7	3.1	5.2
35	42	Cyclopyrimorate	26.6	1.8	2.0	2.7	0.7
36	43	Cyproconazole	6.9	8.9	12.1	3.4	2.9
37	44	Daimuron	9.0	19.0	18.0	16.0	6.1
38	45	Diazinon	3.9	6.6	23.7	14.0	6.2
39	46	Dichlorvos (DDVP)	13.6	17.1	2.3	3.8	6.4
40	47	Diclocymet *E*	2.1	7.3	4.8	36.0	6.6
	48	Diclocymet *Z*	18.7	27.5	17.8	26.1	23.4
41	49	Diflubenzuron	15.1	10.2	6.2	2.9	15.1
42	50	Dimethomorph *E*	11.0	9.7	24.2	5.0	13.6
	51	Dimethomorph *Z*	13.7	18.0	16.1	6.5	20.9
43	52	Dinotefuran	2.4	2.9	5.6	13.7	2.6
44	53	Disulfoton	3.7	1.0	10.1	6.8	6.3
	54	Disulfoton sulfone	0.5	3.5	2.5	3.2	0.2
	55	Disulfoton sulfoxide	2.4	10.6	6.5	5.0	7.6
	56	Demeton-S	2.6	8.8	8.2	20.6	3.0
	57	Demeton-S-sulfone	0.5	12.2	1.5	7.9	3.0
	58	Demeton-S-sulfoxide	0.6	5.7	3.4	0.9	2.4
45	59	Diuron	3.2	11.1	1.3	6.3	8.8
46	60	Edifenphos	2.0	11.7	8.0	13.1	7.4
47	61	Emamectin benzoate	1.1	4.9	5.8	21.0	3.3
48	62	Epoxyconazole	27.2	29.7	26.9	28.1	27.1
49	63	Esprocarb	14.1	3.0	16.1	8.7	0.1
50	64	Ethiofencarb	13.2	15.6	5.9	15.6	1.9
51	65	Ethiprole	8.8	15.2	11.2	5.9	7.5

	66	Ethiprole-sulfone	11.1	24.6	12.5	55.9	11.2
52	67	Ethoxyquin	4.4	21.3	6.8	38.6	2.6
	68	Ethoxyquin dimer	15.8	31.1	12.5	30.4	13.1
53	69	Etobenzanid	4.2	16.0	5.5	5.2	15.6
54	70	Etoxazole	7.5	2.4	5.9	81.3	4.4
55	71	Etrimfos	16.4	6.8	14.5	5.5	5.3
56	72	Famoxadone	1.0	0.6	9.1	1.9	4.0
57	73	Fenamidone	6.9	5.3	8.0	6.4	8.4
58	74	Fenarimol	2.7	0.9	8.8	2.4	7.0
59	75	Fenbuconazole	10.4	2.5	9.2	0.2	14.8
60	76	Fenhexamid	4.1	3.9	5.9	0.9	0.5
61	77	Fenobucarb	11.9	11.9	17.5	13.2	0.8
62	78	Fenoxasulfone	10.2	2.7	13.1	10.7	5.9
63	79	Fenpyroximate	15.9	19.0	14.1	2.9	10.4
64	80	Fensulfothion	12.6	0.4	3.3	2.7	3.2
65	81	Fenthion (MPP)	3.5	9.1	0.0	3.4	1.5
	82	Fenthion oxon sulfone	8.1	10.2	6.3	8.4	2.8
	83	Fenthion oxon sulfoxide	4.6	9.0	6.3	13.3	4.1
	84	Fenthion oxon	0.8	1.3	8.8	4.1	1.6
	85	Fenthion sulfone	1.0	7.7	0.3	7.1	1.7
	86	Fenthion sulfoxide	0.9	20.4	5.9	3.9	3.4
66	87	Fentrazamide	0.6	19.6	8.1	8.0	7.3
67	88	Ferimzone *E*	10.2	6.2	3.7	12.2	0.2
	89	Ferimzone *Z*	3.2	2.7	6.0	8.3	3.2
68	90	Flonicamid	10.1	14.6	0.7	6.1	7.8
	91	TFNA	9.5	17.4	5.5	11.5	9.4
69	92	Florpyrauxifen-benzyl	4.2	1.7	11.0	5.8	2.0
70	93	Fluazinam	4.1	2.3	1.9	1.9	8.9
71	94	Flubendiamide	10.9	24.9	53.5	0.6	3.8
72	95	Fludioxonil	2.4	18.0	1.9	17.5	3.8
73	96	Flufenoxuron	3.2	2.8	11.5	46.0	1.2
74	97	Flumioxazine	2.5	6.5	3.7	16.8	18.1
75	98	Fluopicolide	4.7	17.1	8.2	8.1	3.7
76	99	Flupyrimin	6.4	5.8	10.6	25.1	6.6
77	100	Fluralaner	0.4	9.9	3.9	7.2	5.5
78	101	Fluridone	14.2	4.4	10.6	0.6	8.8

79	102	Flutolanil	17.4	28.1	30.9	20.9	1.2
80	103	Fluxametamide	8.6	13.2	19.1	14.3	18.8
81	104	Fluxapyroxad	8.0	1.5	15.5	16.3	10.6
82	105	Furametpyr	11.8	8.3	11.1	0.4	0.4
83	106	Hexaconazole	4.3	9.7	29.9	6.3	2.6
84	107	Imazalil	13.7	9.8	7.6	6.2	7.1
85	108	Inpyrfluxam	10.5	4.2	14.4	13.0	18.5
86	109	Ipfencarbazone	5.5	6.9	14.3	8.8	15.7
87	110	Ipflufenoquin	18.0	1.3	15.6	7.2	12.0
88	111	Iprobenfos	15.1	12.2	11.0	8.6	1.0
89	112	Isoprothiolane	0.7	17.4	9.3	8.8	9.9
90	113	Isoxathion	22.0	3.0	30.2	7.5	25.7
91	114	Lufenuron	3.5	37.3	29.0	61.9	1.1
92	115	Malathion	18.4	4.9	2.5	12.1	7.9
93	116	Mefenacet	12.5	6.3	21.4	7.5	0.7
94	117	Metaflumizone *E*	4.2	5.7	2.5	7.8	0.1
	118	Metaflumizone *Z*	20.0	5.7	20.8	10.0	0.3
95	119	Metalaxyl	4.1	7.8	6.0	17.1	0.8
96	120	Methamidophos	3.2	62.9	12.8	27.4	12.1
97	121	Methidathion	2.6	3.4	6.5	1.6	0.9
98	122	Methiocarb	0.6	20.3	8.0	16.2	8.0
99	123	Methoxyfenozide	14.0	13.7	19.0	1.6	4.7
100	124	Metominostrobin *E*	7.6	35.5	3.9	9.3	4.5
101	125	Metrafenone	19.0	6.0	9.3	25.3	2.7
102	126	Metyltetraprole	4.8	7.7	12.8	7.1	4.3
103	127	Molinate	1.1	8.2	5.3	13.7	4.9
104	128	Monocrotophos	2.7	6.0	11.0	1.0	16.3
105	129	Myclobutanil	6.3	16.9	7.3	13.9	15.1
106	130	Novaluron	2.4	1.9	7.2	1.2	3.6
107	131	Orysastrobin	5.5	6.0	23.7	1.7	5.6
108	132	Oxadiargyl	5.6	2.3	6.5	2.9	4.2
109	133	Oxathiapiprolin	1.7	8.1	15.3	0.9	8.2
110	134	Oxaziclomefone	8.9	3.0	9.2	10.7	3.9
111	135	Oxydemeton-methyl	17.1	1.3	10.8	6.7	11.9
112	136	Penconazole	11.6	3.4	0.7	2.0	17.5
113	137	Pencycuron	26.9	11.0	5.6	9.2	1.2

114	138	Pendimethalin	12.6	4.6	10.6	7.0	6.5
115	139	Penoxsulam	11.6	17.0	6.0	10.5	13.9
116	140	Phorate	5.8	1.3	1.3	3.6	2.2
	141	Phorate oxon sulfone	7.2	9.3	1.5	6.7	13.2
	142	Phorate oxon sulfoxide	4.3	13.6	2.5	2.0	1.7
	143	Phorate oxon	23.2	11.9	3.1	14.4	4.8
	144	Phorate sulfone	10.4	3.9	4.0	6.5	14.3
	145	Phorate sulfoxide	7.8	12.1	5.1	6.0	2.2
117	146	Phoxim	1.0	9.7	8.0	9.3	10.0
118	147	Pirimicarb	6.6	16.5	1.2	17.4	8.6
119	148	Probenazole	0.2	10.9	3.3	1.5	4.2
120	149	Profenofos	11.2	24.5	5.4	20.7	0.9
121	150	Propamocarb	12.9	6.5	14.9	14.4	11.3
122	151	Propiconazole	4.2	1.6	10.3	4.1	1.8
123	152	Propoxur	0.8	8.3	5.1	15.2	3.8
124	153	Propyrisulfuron	0.2	2.1	1.9	4.4	12.1
125	154	Prosulfocarb	3.8	19.1	13.3	16.7	1.2
126	155	Pydiflumetofen	25.9	12.5	3.4	7.4	3.1
127	156	Pyraclostrobin	10.0	14.3	7.4	10.2	2.6
128	157	Pyribencarb	6.2	11.9	1.1	1.0	7.4
129	158	Pyributicarb	2.2	8.6	20.9	36.8	5.0
130	159	Pyridaben	9.2	13.7	2.1	21.8	1.9
131	160	Pyriofenone	5.7	2.8	3.6	4.7	4.1
132	161	Pyriproxyfen	7.7	5.4	0.7	19.1	15.0
133	162	Pyroquilon	5.5	0.5	8.2	19.7	1.3
134	163	Quinoclamine	7.6	11.1	17.2	7.6	7.5
135	164	Saflufenacil	1.1	2.5	10.5	1.6	8.9
136	165	Sedaxane	5.3	7.3	7.9	3.4	11.0
137	166	Sethoxydim	2.8	13.4	12.1	85.6	11.3
138	167	Simazine	1.7	4.2	11.5	5.0	6.5
	168	Shimazine-2-hydroxy (OH-Simazine)	0.8	5.2	58.8	63.7	10.3
139	169	Spinetoram J	10.2	4.2	18.8	7.8	9.0
	170	Spinetoram L	9.7	2.6	1.4	19.9	4.1
140	171	Spinosyn A	11.4	15.6	7.0	8.7	0.9
	172	Spinosyn D	1.8	17.6	12.5	20.3	5.1

141	173	Spiromesifen	16.4	1.5	3.7	69.6	0.2
142	174	Spirotetramat	16.2	7.4	7.8	5.7	1.2
143	175	Sulfosulfuron	3.3	19.1	16.7	4.9	5.4
144	176	Sulfoxaflor	3.6	11.8	7.2	14.9	4.7
145	177	Tebuconazole	3.0	10.2	4.5	2.3	2.0
146	178	Tebufenozide	2.1	7.0	38.9	9.4	12.3
147	179	Tebufloquin	5.0	4.4	2.7	16.7	2.3
	180	Tebufloquin M1	15.0	7.6	6.8	2.5	1.0
148	181	Teflubenzuron	7.3	35.1	39.6	61.1	1.5
149	182	Terbuthylazine	9.0	8.5	3.3	14.8	8.2
	183	Terbuthylazine-2-hydroxy (OH-TER)	3.7	10.7	8.7	3.3	1.9
	184	Terbuthylazine-desethyl (DE-TER)	3.8	9.4	3.8	18.5	7.6
	185	Terbuthylazine-desethyl-2-hydroxy (OH-DE-TER)	9.7	15.3	53.1	54.4	2.5
150	186	Tetraniliprole	2.1	14.2	27.1	21.0	12.6
151	187	Thiabendazole	7.0	14.4	3.4	2.3	5.2
	188	5-Hydroxy thiabendazole	1.3	13.3	15.8	10.1	2.0
152	189	Thiacloprid	5.4	15.4	2.3	9.7	4.5
153	190	Thiamethoxam	3.4	10.3	0.1	4.2	1.4
154	191	Thiobencarb	4.2	12.0	1.5	31.5	2.0
155	192	Tiadinil	2.4	11.7	10.7	14.8	7.3
156	193	Tolprocarb	10.0	9.1	9.4	2.3	3.2
157	194	Trichlorfon (Metrifonate)	18.4	18.1	4.4	22.5	12.5
158	195	Tricyclazole	3.8	25.8	1.3	8.9	4.5
159	196	Trifloxystrobin	3.6	13.1	3.7	25.0	2.0
160	197	Triflumezopyrim	6.3	2.5	0.6	0.9	4.0
161	198	Warfarin	8.4	11.0	9.3	21.1	11.2

Table S5. Acceptable daily intake (ADI) values for all analyzed compounds

No.	Pesticide	Acceptable daily intake (mg/kg・BW /day)	Reference
1	Acephate	0.03	MFDS
2	Acetamiprid	0.071	MFDS
3	Acynonapyr	0.04	MFDS
4	Alachlor	0.01	MFDS
5	Aldicarb	0.003	MFDS
6	Amitraz	0.01	MFDS
7	Atrazine	0.02	EFSA, WHO
8	Azinphos-methyl	0.03	WHO
9	Azoxystrobin	0.2	MFDS
10	Bendiocarb	0.004	MFDS
11	Bensulfuron methyl	0.2	EFSA
12	Benzovindiflupyr	0.049	MFDS
13	Benzpyrimoxan	0.1	WHO
14	Bifenazate	0.01	MFDS
15	Bioresmethrin	0.03	MFDS
16	Boscalid	0.04	MFDS
17	Brodifacoum	-	-
18	Buprofezin	0.01	MFDS
19	Butamifos	0.008	FSCJ
20	Cafenstrole	0.003	MFDS
21	Carbaryl	0.0075	MFDS
22	Carbendazim	0.03	MFDS
23	Carbofuran	0.001	MFDS
24	Carbosulfan	0.001	MFDS
25	Carfentrazone-ethyl	0.03	MFDS
26	Carpropamide	0.014	MFDS
27	Chlorantraniliprole	2	MFDS
28	Chlorfenvinphos	0.0005	MFDS
29	Chromafenozide	0.27	MFDS
30	Clofentezine	0.017	MFDS
31	Clomeprop	0.0062	RDA
32	Clothianidin	0.097	MFDS
33	Cumyluron	-	-
34	Cyantraniliprole	0.057	MFDS
35	Cyclopyrimorate	0.063	FSCJ
36	Cyproconazole	0.02	MFDS
37	Daimuron	0.3	MFDS

38	Diazinon	0.0002	MFDS
39	Dichlorvos (DDVP)	0.004	MFDS
40	Diclocymet	-	-
41	Diflubenzuron	0.02	MFDS
42	Dimethomorph	0.2	MFDS
43	Dinotefuran	0.02	MFDS
44	Disulfoton	0.00004	MFDS
45	Diuron	0.007	MFDS
46	Edifenphos	0.003	MFDS
47	Emamectin benzoate	0.0025	MFDS
48	Epoxiconazole	0.007	MFDS
49	Esprocarb	0.01	MFDS
50	Ethiofencarb	0.1	MFDS
51	Ethiprole	0.005	MFDS
52	Ethoxyquin	0.005	MFDS
53	Etobenzanid	-	-
54	Etoxazole	0.04	MFDS
55	Etrimfos	0.003	MFDS
56	Famoxadone	0.006	MFDS
57	Fenamidone	0.028	MFDS
58	Fenarimol	0.01	MFDS
59	Fenbuconazole	0.03	MFDS
60	Fenhexamid	0.2	MFDS
61	Fenobucarb	0.014	MFDS
62	Fenoxasulfone	0.018	MFDS
63	Fenpyroximate	0.01	MFDS
64	Fensulfothion	0.0003	MFDS
65	Fenthion (MPP)	0.007	WHO
66	Fentrazamide	0.0052	MFDS
67	Ferimzone	0.019	MFDS
68	Flonicamid	0.025	MFDS
69	Florpyrauxifen-benzyl	2.4	MFDS
70	Fluazinam	0.01	MFDS
71	Flubendiamide	0.017	MFDS
72	Fludioxonil	0.4	MFDS
73	Flufenoxuron	0.037	MFDS
74	Flumioxazin	0.02	WHO
75	Fluopicolide	0.079	MFDS
76	Flupyrimin	0.01	MFDS
77	Fluralaner	0.01	MFDS
78	Fluridone	0.08	EPA
79	Flutolanil	0.09	MFDS

80	Fluxametamide	0.0085	MFDS
81	Fluxapyroxad	0.021	MFDS
82	Furametpyr	-	-
83	Hexaconazole	0.005	MFDS
84	Imazalil	0.03	MFDS
82	Inpyrfluxam	0.06	WHO
86	Ipfencarbazone	0.001	MFDS
87	Ipflufenoquin	0.05	MFDS
88	Iprobenfos	0.035	MFDS
89	Isoprothiolane	0.1	MFDS
90	Isoxathion	0.02	FSCJ
91	Lufenuron	0.015	MFDS
92	Malathion	0.029	MFDS
93	Mefenacet	0.007	MFDS
94	Metaflumizone	0.1	MFDS
95	Metalaxyl	0.08	MFDS
96	Methamidophos	0.004	MFDS
97	Methidathion	0.001	MFDS
98	Methiocarb	0.02	MFDS
99	Methoxyfenozide	0.1	MFDS
100	Metominostrobin	0.016	FSCJ
101	Metrafenone	0.25	MFDS
102	Metyltetraprole	2.5	FSCJ
103	Molinate	0.0021	MFDS
104	Monocrotophos	0.0006	MFDS
105	Myclobutanil	0.03	MFDS
106	Novaluron	0.01	MFDS
107	Orysastrobin	0.052	MFDS
108	Oxadiargyl	0.008	MFDS
109	Oxathiapiprolin	1.04	MFDS
110	Oxaziclomefone	0.0091	MFDS
111	Oxydemeton-methyl	0.0003	EFSA
112	Penconazole	0.03	MFDS
113	Pencycuron	0.2	MFDS
114	Pendimethalin	0.13	MFDS
115	Penoxsulam	0.05	MFDS
116	Phorate	0.0007	MFDS
117	Phoxim	0.004	MFDS
118	Pirimicarb	0.02	MFDS
119	Probenazole	0.01	MFDS
120	Profenofos	0.03	MFDS
121	Propamocarb	0.4	MFDS

122	Propiconazole	0.07	MFDS
123	Propoxur	0.005	MFDS
124	Propyrisulfuron	0.011	MFDS
125	Prosulfocarb	0.005	EFSA
126	Pydiflumetofen	0.092	MFDS
127	Pyraclostrobin	0.03	MFDS
128	Pyribencarb	0.039	MFDS
129	Pyributicarb	0.0088	MFDS
130	Pyridaben	0.005	MFDS
131	Pyriofenone	0.091	MFDS
132	Pyriproxyfen	0.1	MFDS
133	Pyroquilon	0.019	FSCJ
134	Quinoclamine	0.0021	MFDS
135	Saflufenacil	0.046	MFDS
136	Sedaxane	0.11	MFDS
137	Sethoxydim	0.14	MFDS
138	Simazine	0.018	MFDS
139	Spinetoram	0.05	MFDS
140	Spinosyn A	0.02	MFDS
141	Spiromesifen	0.03	MFDS
142	Spirotetramat	0.05	MFDS
143	Sulfosulfuron	0.24	EFSA
144	Sulfoxaflor	0.05	MFDS
145	Tebuconazole	0.03	MFDS
146	Tebufenozide	0.02	MFDS
147	Tebufloquin	0.041	MFDS
148	Teflubenzuron	0.01	MFDS
149	Terbuthylazine	0.004	EFSA
150	Tetraniliprole	0.88	MFDS
151	Thiabendazole	0.1	MFDS
152	Thiacloprid	0.01	MFDS
153	Thiamethoxam	0.08	MFDS
154	Thiobencarb	0.009	MFDS
155	Tiadinil	0.04	MFDS
156	Tolprocarb	-	-
157	Trichlorfon (Metrifonate)	0.002	WHO
158	Tricyclazole	0.05	MFDS
159	Trifloxystrobin	0.04	MFDS
160	Triflumezopyrim	0.12	MFDS
161	Warfarin	0.0003	EPA
